# Research Advances in Superabsorbent Polymers

**DOI:** 10.3390/polym16040501

**Published:** 2024-02-12

**Authors:** Yaoyao Yang, Zhiyuan Liang, Rui Zhang, Shengwei Zhou, Haobo Yang, Yanyu Chen, Jiahui Zhang, Hongyi Yin, Dengguang Yu

**Affiliations:** School of Materials and Chemistry, University of Shanghai for Science and Technology, 516 Jungong Road, Shanghai 200093, China; 212203084@st.usst.edu.cn (Z.L.); 212203081@st.usst.edu.cn (R.Z.); 213353182@st.usst.edu.cn (S.Z.); 2135054023@st.usst.edu.cn (H.Y.); 2135054608@st.usst.edu.cn (Y.C.); 2135054528@st.usst.edu.cn (J.Z.); 2135054424@st.usst.edu.cn (H.Y.)

**Keywords:** superabsorbent polymers, polyacrylic acid, hydrogel, nanofiber, nanomedicine

## Abstract

Superabsorbent polymers are new functional polymeric materials that can absorb and retain liquids thousands of times their masses. This paper reviews the synthesis and modification methods of different superabsorbent polymers, summarizes the processing methods for different forms of superabsorbent polymers, and organizes the applications and research progress of superabsorbent polymers in industrial, agricultural, and biomedical industries. Synthetic polymers like polyacrylic acid, polyacrylamide, polyacrylonitrile, and polyvinyl alcohol exhibit superior water absorption properties compared to natural polymers such as cellulose, chitosan, and starch, but they also do not degrade easily. Consequently, it is often necessary to modify synthetic polymers or graft superabsorbent functional groups onto natural polymers, and then crosslink them to balance the properties of material. Compared to the widely used superabsorbent nanoparticles, research on superabsorbent fibers and gels is on the rise, and they are particularly notable in biomedical fields like drug delivery, wound dressing, and tissue engineering.

## 1. Introduction

Superabsorbent polymeric materials were first synthesized by employing acrylic acid (AA) and divinylbenzene in aqueous solutions in the 1840s [1]. In the 1860s, Fanta et al. grafted acrylonitrile onto starch. Subsequent research revealed that superabsorbent polymers derived from starch exhibit exceptional water absorption capabilities and retain their properties when subjected to pressure [2,3,4]. In the 1870s, scientists developed superabsorbent polymers by grafting hydrolyzed acrylonitrile onto starch molecules to help plants grow in desert environments [5]. In 1978, superabsorbent polymers were commercially produced in Japan for use in feminine products. This innovation has enabled the production of more efficient and effective feminine hygiene products. This technology was soon adopted by other countries and has since become an industrial standard. In the 1880s, France and Germany developed superabsorbent polymers synthesized from polyacrylic acid (PAA) blended with cellulose-based fillers. The resulting product was utilized in baby diapers, which increased their absorbency and minimized the quantity of superabsorbent polymers required [6]. After ARCO Chemical developed a highly absorbent fiber in 1890s, and the USDA developed starch-grafted crosslinked polyacrylate polymers that could absorb more than 400 times their own weight in liquid, polyacrylate-based superabsorbent polymers quickly captured a large share of the market [7,8]. Although PAA-based superabsorbent polymers exhibit excellent properties, they are not biodegradable. As concerns about plastic pollution continue to grow worldwide, researchers have begun to focus on the variety of natural superabsorbent polymers, often considered green, that achieve superabsorbent properties by grafting AA onto the polymer backbone [9,10,11].

Superabsorbent polymers are three-dimensional organic materials with moderately crosslinked structures that swell in aqueous solutions, absorb more than 1000 times their dry weight in liquids, and do not release absorbed liquid under pressure [12,13]. The main reason for the water absorption is the osmotic pressure and the presence of numerous hydrophilic functional groups in the molecular chain [14,15], Their water absorption mechanism follows Flory’s network theory, in which the osmotic pressure difference drives the water absorption and expansion [16]. Water absorption by superabsorbent polymers is a physicochemical process, in which the capillary adsorption and polymer network first absorbs part of the water and in a short time reaches the saturation state of physical adsorption. Subsequently, due to the influence of water, the ionic groups within the polymer chain transform into anions and cations. This transformation, coupled with the electrostatic interactions between these ions, causes the molecular chain to expand and unfurl. At this stage, the ion concentration within the polymer is significantly greater than that outside, leading to an osmotic pressure that drives water from the exterior to penetrate the interior until an equilibrium is reached between the internal and external osmotic pressures. The duration of this process will be longer than the physical adsorption. Simultaneously, the polymer chemical water absorption and swelling reaches equilibrium and can be maintained under a certain pressure for a considerable period. The water absorbency of superabsorbent polymers is typically influenced by the pH and ionic concentration of the solution [17]. An increase in the ionic concentration results in a significant decrease in the water absorption capacity and to a greater extent in the case of multivalent cations. This may be because of the chelating effect of the multivalent cations with the polymer functional groups, causing the loosely packed polymer network to become tightly packed through ionic crosslinking and making it extremely difficult for water to re-enter [18,19]. In a strongly acidic environment, H+ replaces cations in the molecular chain, causing the osmotic pressure difference to be greatly reduced. More hydrogen bonds are formed, causing the network structure to become more compact, limiting the movement and relaxation of the molecular chain, and leading to less water absorption. As pH increases, ionization is enhanced, causing the breaking of hydrogen bonds, electrostatic repulsion between polymer chains, and expansion of the polymer network structure, allowing more water to enter. As the water absorption increases, the osmotic pressure decreases, the traction between the polymer networks gradually increases, the water absorption rate reaches equilibrium and stabilizes, and any further increase in pH leads to the structural disintegration of the polymer networks, which will leads to a decrease in water absorption capacity.

Superabsorbent polymers are widely used in various fields owing to their distinctive characteristics [20]. In agriculture, superabsorbent polymers can be used to control the release of water, fertilizers, pesticides, herbicides, etc., and to protect the soil from the impacts that cause it to harden [21,22]. Industrial applications include the adsorption and filtration of toxic heavy metals or dyes in industrial wastewater [23], treatment of radioactive uranium ions [24], dewatering of coal [25], and food packaging [26]. In the biomedical field, superabsorbent polymers are used in tissue engineering [27], biosensing [28], drug delivery systems, and wound dressings [29,30,31,32,33,34,35]. Superabsorbent polymers also play a significant role in secondary energy storage, thermal energy storage, and artificial snowmaking [36,37,38]. The most commonly used superabsorbent polymers and their applications are listed in Table 1, along with the corresponding physical and chemical properties and water absorption ability of the materials.

This article will provide a comprehensive overview of existing studies on superabsorbent polymers, focusing on their types, morphology, and applications. The article delves into the multifunctional applications of superabsorbent polymers while addressing the relationship between composition, structure, and performance of superabsorbent polymer materials, offering insights into new design strategies for innovative functional materials.

## 2. Superabsorbent Polymers

### 2.1. Synthetic Superabsorbent Polymers

There are two major categories of superabsorbent polymers: synthetic and natural. Synthetic polymers include PAA [67], polyacrylamide [68], polyacrylonitrile [69], and polyvinyl alcohol (PVA) [70].

#### 2.1.1. Polyacrylic Acids

**Figure 1:**
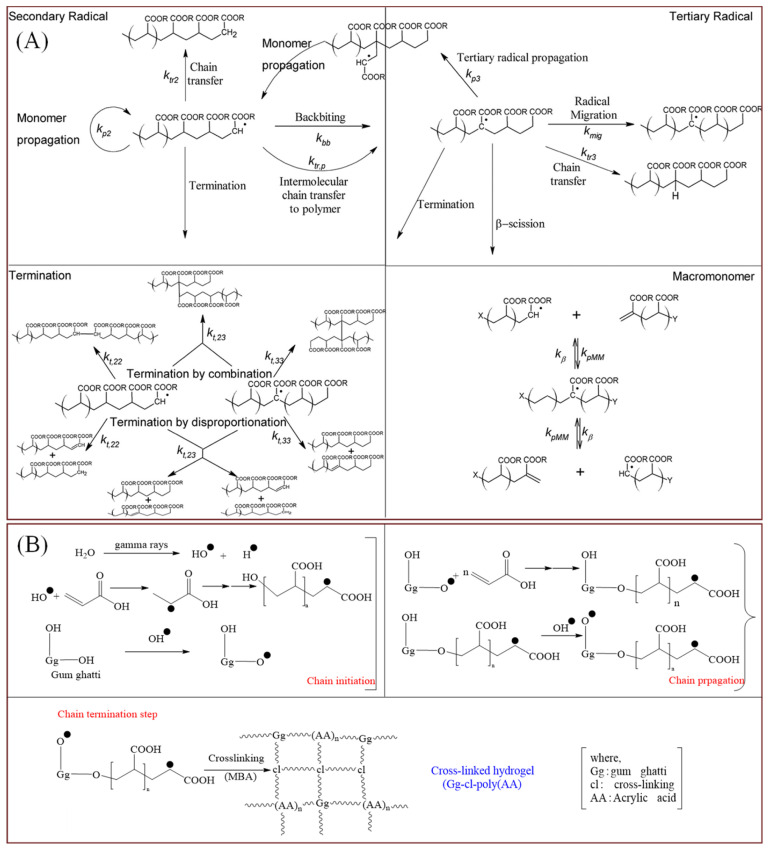
(**A**) Potential reactions during free radical polymerization of acrylates [71]. Copyright 2018, reproduced with permission of Elsevier. (**B**) Acrylic Graft Copolymerization [72]. Copyright 2022, reproduced with permission of Elsevier.

PAA is the most commonly used superabsorbent. Potassium acrylate and acrylamide can polymerize and crosslink to form acrylic superabsorbent polymer network structures under the action of the initiator potassium persulfate (K_2_S_2_O_8_) and the crosslinker N, N-methylene bisacrylamide (MBA). Various potential reaction principles occur in the free radical polymerization of acrylates, as shown in Figure 1A [71]. Figure 1B illustrates the reaction mechanism for the formation of crosslinked acrylic hydrogels via radiation-induced graft polymerization [72]. The molecular weight of the crosslinked PAA main chain can reach 10^7^ g/mol, and the content of water-absorbing groups is much higher than that of other polymer materials, such as polyvinyl alcohol and modified polyacrylonitrile, which provides a very high water-absorbing and long-lasting water-retaining capacity [73]. Acrylics dominate the superabsorbent market owing to their relatively inexpensive raw materials and ease of synthesis from high-molecular-weight products [74].

**Figure 2:**
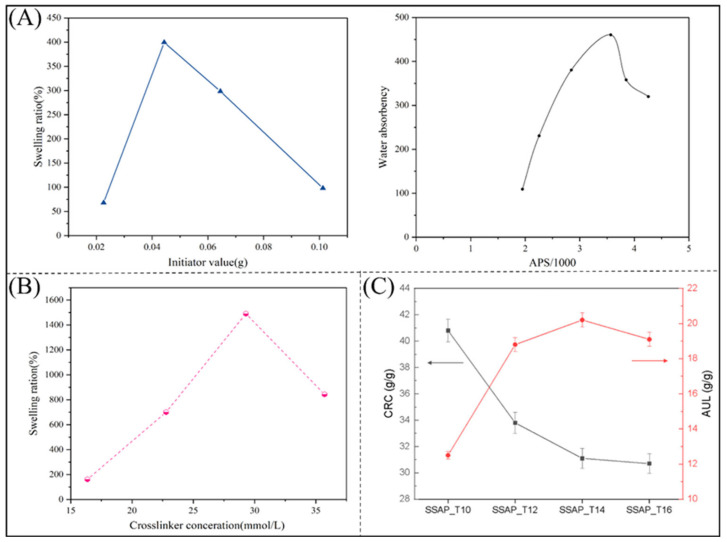
(**A**) Effect of initiator dose on polymer swelling ratio [75,76]. Copyright 2021, reproduced with permission of John Wiley and Sons. Copyright 2002, reproduced with permission of Elsevier. (**B**) Effect of crosslinker content on polymer water absorption [78]. Copyright 2018, reproduced with permission of Elsevier (**C**) Effect of crosslinking at different Temperatures on CRC and AUL of polymers [79]. Copyright 2021, reproduced with permission of MDPI.

The type and amount of initiator used in the synthesis process have a strong influence on the water absorption properties of polyacrylate-type polymers. When benzoyl peroxide is used as the initiator, the water absorption of PAA, PVA/AA copolymers, and carboxymethyl cellulose (CMC)/AA copolymers increase with increasing amounts of the initiator. Other studies have shown that when ammonium persulfate is used as an initiator, the water absorption rate first increases and then decreases with increasing initiator content (Figure 2A) [75,76]. The water absorption rate of the material increases with increasing dosage at a low initiator dosage when the conversion rate of the monomer is high; an initiator dosage that is too high will increase the collision of monomer free radicals, increasing the soluble portion of the polymer and decreasing the water absorption capacity [77].

Figure 2B shows the effect of different amounts of crosslinker on the water absorption rate [78]. The water absorption rate of the superabsorbent polymers tended to increase and then decrease with increasing crosslinker content. When the crosslinker content was low, the reaction system could not form a desirable three-dimensional crosslinked network, and some monomers did not form crosslinks, resulting in a lower water absorption rate. When the content of crosslinking agent is too high, the crosslinking density is also too high, and although the molecular chain can expand smoothly, the intermolecular space is too small for water molecules to enter; thus, the water absorption of the superabsorbent polymers decreases.

Figure 2C shows the centrifuge retention capacity (CRC) and absorption under load (AUL) of water-absorbent polymers after the crosslinking reaction at different temperatures [79]. The CRC of the polymers gradually decreases as the reaction temperature increases; however, the AUL shows a different trend, increasing with the reaction temperature but decreasing again at 160 °C. This is mainly due to the dehydration of the carboxylic acid groups at 160 °C [80].

**Figure 3:**
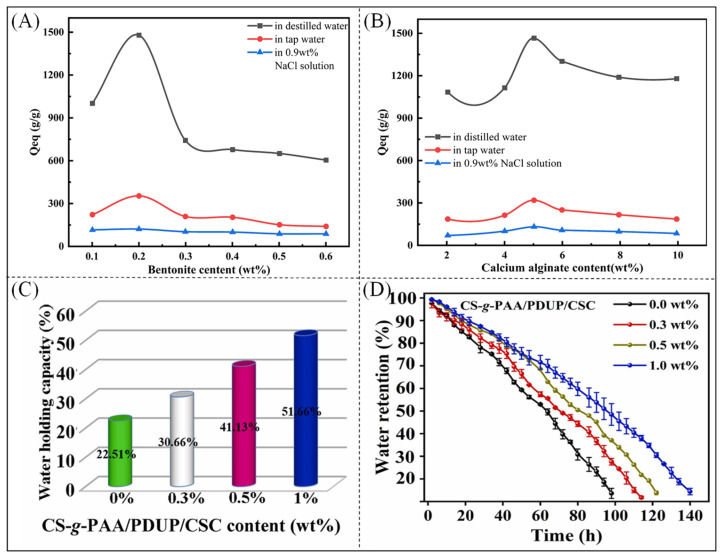
(**A**) Effect of sodium bentonite content on water absorption of materials. (**B**) Effect of calcium alginate content on water absorption of polymers [55]. Copyright 2022, reproduced with permission of Elsevier Effect of semi-tar shale addition on soil (**C**) water content and (**D**) water retention [84]. Copyright 2022, reproduced with permission of Elsevier.

Because of the high stability, very high molecular weight, and low water solubility of polyacrylates, which result in the inability of microorganisms to degrade them and a tendency to cause environmental pollution problems, the materials need to be modified [81]. The most common method is to add inorganic additives to the material to improve solubility and strength [82,83]. As shown in Figure 3A, the swelling rate of the superabsorbent composites was highest at a sodium bentonite content of 2 wt% and gradually decreased as the content of sodium bentonite increased to 8 wt%. This is because the number of water molecules that could enter the pores decreased, and the excess sodium bentonite filled the internal space of the polymer and reduced the proportion of hydrophilic groups per unit volume. Figure 3B shows the relationship between the content of calcium alginate, another inorganic additive, and the water absorption rate of the superabsorbent composite. The addition of an appropriate amount of calcium alginate increases the number of hydrophilic groups in the polymer network and improves the water absorption rate [55]. As shown in Figure 3C,D, when superabsorbent composites with different proportions of semi-tar shale were added to soil, the water-holding capacity of the superabsorbent materials and the water retention rate of the soil gradually increased with an increase in the content of semi-tar shale [84].

#### 2.1.2. Polyacrylonitrile

PAA-based superabsorbent polymers can agglomerate after absorbing water, resulting in hindered water absorption. Thus, polyacrylonitrile fiber superabsorbent materials were developed in the 1980s; fibers are mainly produced by spinning or stretching processes, and after post-processing, polyacrylonitrile fibers can be obtained with the desired properties. Because the nitrile group in polyacrylonitrile is electronegative and cannot easily absorb water, it must be hydrolyzed by adding alkali to change the hydrophobic group into an alkali metal carboxylic acid group to obtain the ability to absorb water, after which the hydrophilic group in the three-dimensional network locks the water into the pores of the network by hydrogen bonding. Using polyacrylonitrile semi-finished fibers as raw material for alkaline hydrolysis reaction, and then through esterification in glycerol aqueous solution, super-absorbent polyacrylonitrile fibers can be prepared; the water absorption rate reaches 42 g distilled water/g fiber. Again, the water absorption is good, but the mechanical properties of the fiber will be slightly reduced [85]. The effects of alkali concentration, hydrolysis time, and hydrolysis temperature on the water absorption rate are similar. When the alkali concentration is low or the hydrolysis time is short, the degree of hydrolysis of the fiber is relatively low after hydrolysis, and the number of hydrophilic groups will be relatively small if the water absorption of the crosslinked network structure does not open easily, thus leading to a low water absorption rate. An appropriate increase in alkali concentration, hydrolysis time, and hydrolysis temperature may result in complete hydrolysis of the nitrile group [86]. Hydrolysis changes the chemical structure and surface structure of the fiber, resulting in the appearance of a loose porous fiber surface phenomenon, further increasing the contact area of the fiber and water. Improving the fiber water absorption rate and water absorption multiplier has a promoting effect, but will lead to a decrease in mechanical properties [87].

#### 2.1.3. Polyvinyl Alcohol

**Figure 4:**
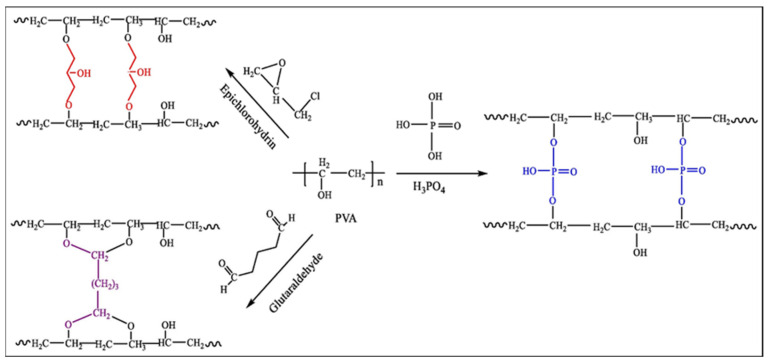
Polyvinyl alcohol crosslinking reaction [72]. Copyright 2022, reproduced with permission of Elsevier.

PVA is not as absorbent as PAA-based superabsorbent polymers but is biodegradable. PVA is a hydrophilic polymer with multiple hydroxyl groups, which have good water absorption and water retention capacities after crosslinking. PVA becomes a non-toxic, colorless, odorless, and transparent solution after dissolving in water because it is water-soluble. However, it cannot absorb large amounts of water, and carboxyl groups can be introduced into PVA molecules by direct chemical modification of the super-absorbent fibers. Superabsorbent PVA fibers have a large specific surface area, and the existence of a variety of hydrophilic groups in the molecule leads to its hydrophilicity being very strong and having low crystallinity; in the reaction of a low molecular crosslinking agent into the amorphous region and the hydroxyl group in the PVA crosslinking grafting reaction, a crosslinked network structure is formed. PVA has certain processing abilities, including pH strength, temperature stability, biocompatibility, and non-toxic biodegradability, and in the field of pharmaceuticals, it has a wide range of applications [88]. Figure 4 summarizes some of the crosslinking reactions of PVA [72].

### 2.2. Natural Superabsorbent Polymer

In recent years, natural polymers, especially polysaccharide-based superabsorbent polymers, have garnered significant attention due to their non-toxicity and good hydrophilicity, biocompatibility, and biodegradability compared with synthetic polymers. The main materials used for superabsorbent polymers are natural polymers, such as cellulose [48,49,50,89], chitosan [51,52,53,90], starch [54,55,56,57,91], proteins [9,58,59,60], amino acids [61,62,63,92], and alginate [64,65,66,93].

#### 2.2.1. Common Natural Polymers

**Figure 5:**
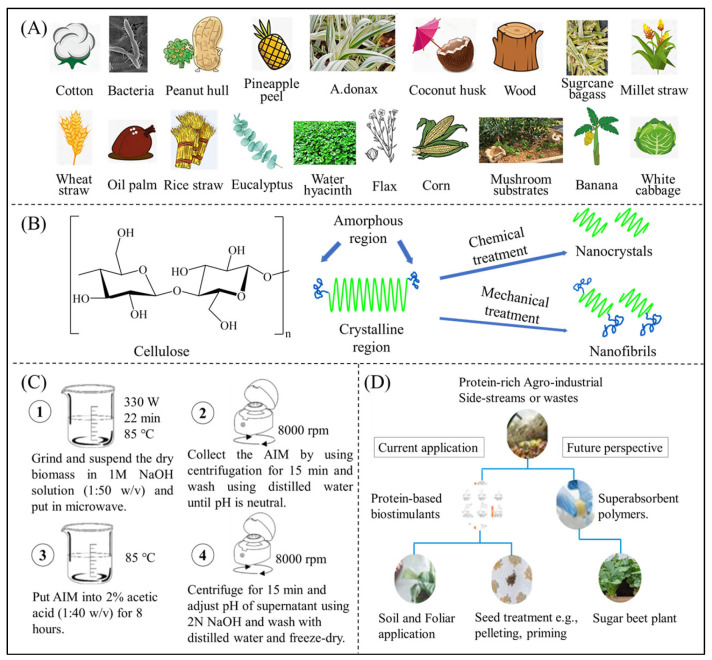
(**A**) Cellulose raw materials [94]. (**B**) Cellulose to nanocellulose processing [94]. Copyright 2023, reproduced with permission of Elsevier. (**C**) Chitosan extraction process [96] Copyright 2022, reproduced with permission of Elsevier. (**D**) Protein-based bio stimulants [97]. Copyright 2022, reproduced with permission of MDPI.

Natural superabsorbent polymers usually require extraction from various raw materials via various means and crosslinking treatments to form a network structure. Cellulose is widely available in nature, as shown in Figure 5A, which shows the various raw plant materials that can be used to extract cellulose in order of their cellulose content [94]. In the industrial field, different cellulose and its derivatives can be obtained through different extraction processes. Generally, the more polar hydrophilic groups the cellulose and its derivatives contain, the stronger their water absorption performance becomes. A variety of bacteria and fungi exist in nature that can secrete cellulase enzymes to degrade cellulose. Thus, cellulose-based materials are considered environmentally friendly substitutes for synthetic polymers. Cellulose usually exists in the form of fibrous strands with a large number of intramolecular and intermolecular hydrogen bonds. The fibrous strands are converted into cellulose nanofibers and nanocrystals by physicochemical treatments with abundant hydroxyl groups for crosslinking (Figure 5B). Sodium or potassium salts are usually synthesized when CMC is used to obtain better water absorption, whereas the addition of HEC promotes intermolecular crosslinking, thus improving the poor crosslinking properties of CMC. Cellulose can also be crosslinked with other polysaccharides via irradiation or chemical crosslinking agents [95].

Starch, the most prevalent polysaccharide in plants [98], has two main structures, straight-chain starch and branched-chain starch [99], both of which are readily hydrolyzed by enzymes or microorganisms into glucose, which is then metabolized into carbon dioxide and water [100]. Starch is rich in hydroxyl groups; therefore, it can be used to prepare superabsorbent materials, is widely available, is easy to modify at low cost, and has good processing properties. There are two main methods for preparing polysaccharide-based superabsorbent polymers: (1) direct crosslinking of polysaccharides and (2) grafting vinyl monomers onto polysaccharides and then crosslinking to form a network structure.

Chitosan is the second most abundant naturally occurring macromolecule after cellulose [101], has a linear polysaccharide structure, and is commonly found in fungi, arthropod exoskeletons, and molluscan teeth [102]. The general chitosan extraction process is shown in Figure 5C [96]. Due to their strong hydrogen bonding, chitosan molecules exhibit poor solubility, whereas derivatives such as carboxymethyl chitosan and quaternary ammonium salt chitosan demonstrate better solubility [103]. Chitosan can be degraded by lysozyme and is also considered an environmentally friendly polymer [104,105]. Chen et al. [106] prepared a novel superabsorbent polymer by grafting copolymerization of sodium acrylate and 1-vinyl-2-pyrrolidone onto the chain of N,O-carboxymethyl chitosan; the measured water absorbency was 1268 g distilled water/g. Quaternary ammonium salt chitosan exhibits excellent antimicrobial properties [107].

The molecular weights and amino acid compositions of different proteins exhibit significant variations [108]. The carboxyl and hydroxyl groups in the molecule can be used to regulate hydrophilicity [109]. Protein materials are widely used in the field of biomedicine [110]. Figure 5D shows some existing applications of protein superabsorbent materials and their prospects for future applications [97]. Collagen, gelatine, and oleoresin are commonly used to produce polymeric superabsorbent materials.

Amino acids are used as monomer units to form polymers with hydrophilic groups that can absorb water, and polyamino acids can be directly crosslinked to obtain a network structure. Materials prepared by crosslinking exhibit excellent water absorption properties. The maximum water absorption capacity of polyaspartic acid can reach 1100 g distilled water/g [111]. Kunioka [112] crosslinked polyglutamic acid using a high dose of γ-radiation and obtained a product with an excellent water absorption capacity (3500 g distilled water/g) but very poor mechanical properties, in addition to the fact that the production of this material, although biodegradable, has not been studied.

Alginates are naturally occurring anionic polymers that are widely used in biomedical and industrial applications owing to their good biocompatibility, low toxicity, and low cost [113]. As the most commonly used type of alginate, sodium alginate has a large number of hydrophilic groups and is suitable for use as a wound dressing material [114]. The carboxyl groups on the alginate backbone can be crosslinked by divalent metal ions, or a stabilizing network structure can be created using a crosslinking agent after graft copolymerization with vinyl monomers. The water absorption properties of alginate-based materials exhibit significant variations [115,116], ranging from dozens to thousands, yet they demonstrate commendable biodegradability [117].

#### 2.2.2. Modified Natural Superabsorbent Polymer

Natural superabsorbent polymers have lower water absorption properties than synthetic superabsorbent polymers, although they are more environmentally friendly. Therefore, the modification of natural superabsorbent polymers has been developed to obtain higher water absorption properties, and existing modification methods usually involve graft copolymerization of polymers rich in hydrophilic groups.

**Figure 6:**
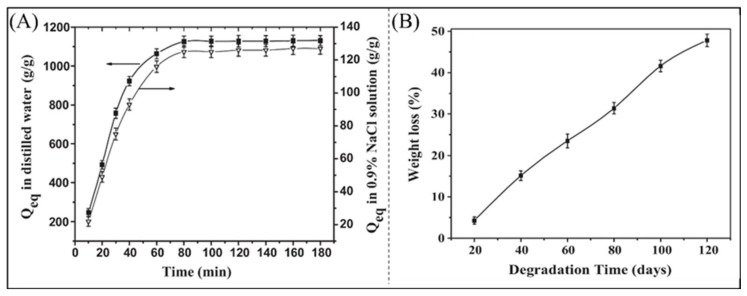
Dissolution kinetics of superabsorbent polymers (**A**) in distilled water and 0.9% NaCl solution; (**B**) degradability in soil burial [120]. Copyright 2014, reproduced with permission of Elsevier.

Acrylic polymers are often grafted onto cellulose to enhance its water absorption capability while preserving its biodegradability [118,119]. As shown in Figure 6, the superabsorbent polymer prepared from AA and alkylated cotton stalk cellulose exhibits an excellent absorption capacity of 1125 g distilled water/g. A biodegradation test in soil with 20% *w/w* moisture showed 46.7% weight loss of the material after 150 days [120]. By grafting acrylic acid or polyacrylamide onto starch, its water absorption capability can be enhanced [121,122,123].

The amino groups on chitosan are less hydrophilic than the carboxyl or hydroxyl groups on cellulose and starch [124], and thus the water absorption capacity of pure chitosan is only 0.9 g distilled water/g. Therefore, physical or chemical modifications are required to improve the water absorption capacities of chitosan and its derivatives. Wang et al. [125] prepared chitosan films with interconnected pores via a ternary solvent system, which improved the water absorption capacity physically. Additionally, the presence of amino groups on the chitosan molecule facilitates the introduction of additional hydrophilic vinyl monomers via graft copolymerization under reactive conditions [126]. Most chitosan-based superabsorbent polymers are chemically modified by introducing hydrophilic groups and constructing crosslinked network [127]. Tang et al. [128] reported a material made of chitosan and carboxymethylcellulose with epichlorohydrin as the crosslinking agent, which had a water absorption capacity of 1300 g of distilled water/g and could be degraded in soil. For proteins with low water absorption [129], the most effective method of modification remains the introduction of hydrophilic acrylic acid units [130]. Pourjavadi et al. [131] grafted AA and AM onto a collagen matrix, followed by further crosslinking, and the composite exhibited high water retention capacity under load. In addition, the absorption capacity of proteins can be improved by chemical functionalization [132,133].

## 3. Forms of Superabsorbent Polymers

Superabsorbent polymer materials are usually prepared in different forms, depending on the application scenario. Superabsorbent polymer particles are the most common and widely used form and are generally prepared by solution or emulsion polymerization, lyophilization, or spray-drying. They can be used in cosmetics, agricultural water retention materials, and industrial adsorbents. Superabsorbent polymers can also be prepared in fiber form by spinning or melt-stretching and can be used in medical dressings, hygiene products, filtration materials, etc. [134,135,136]. Superabsorbent polymer gels are usually polymerized by ionic monomers and are slightly crosslinked. Dry gels or aerogels can be obtained by removing aqueous substances by various means and can be used in hygiene products, drug delivery, water collection, and water purification. In addition, superabsorbent polymers can be produced in the form of films via solution casting and melt-calendaring for use as waterproofing and packaging materials.

### 3.1. Superabsorbent Polymer Particles

**Figure 7:**
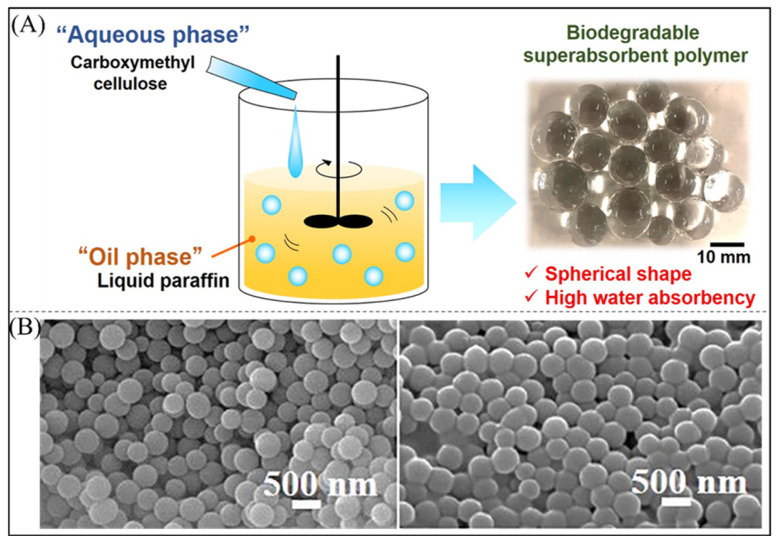
(**A**) Schematic of the synthesis of superabsorbent polymer particles [137]. Copyright 2022, reproduced with permission of MDPI. (**B**) Superabsorbent core-shell particles without crosslinker (**left**) and with 0.5% crosslinker (**right**) [138]. Copyright 2020, reproduced with permission of John Wiley and Sons.

As shown in Figure 7A, Fujita et al. [137] prepared superabsorbent polymer particles that could be used as air fresheners and horticultural water retention agents, using CMC as the raw material and ethylene glycol diglycidyl ether as the crosslinking agent. Maijan et al. [138] prepared a highly absorbent material by the radical polymerization of acrylamide via doping of vinyl-functionalized silica core-shell particles (Figure 7B), which increased the thickness of the core layer and caused agglomeration of the particles. The size of superabsorbent polymer particles has been extensively studied to increase their ultimate water absorption capacity. Yang et al. found that samples with larger agglomerate sizes had higher water absorption rates, but samples with smaller aggregate sizes took less time to reach water absorption equilibrium [139]. Arpit Sand [140] studied the effect of reaction parameters on the water absorption of itaconic acid particles during the synthesis of itaconic acid by reverse suspension polymerization. The viscosity of the organic medium significantly influenced the shape and absorbency of the prepared particles. A viscosity that is too low results in irregularly shaped particles, while an excessively high viscosity results in an unstable suspension with lower water absorption. The addition of a surfactant also has an effect on particle size and water absorption; increasing the concentration of the active agent decreases the particle diameter, and smaller particles increase the surface area, which increases the water absorption. However, too high a concentration decreases the water absorption when the hydrophilic part of the surfactant undergoes chain transfer, resulting in a low molecular weight polymer, and the hydrophobic part prevents water penetration. In general, the higher the mixing speed, the more uniform the particle size distribution to achieve higher absorption, and excessive shear leads to the degradation of the polymer chains’ ability to reduce water absorption.

The absorption capacity and rate of superabsorbent nanoparticles are related to chemical factors such as composition of monomer, degree or type of crosslinking, and polymerization process, which are also affected by particle size [141] and aggregate size [142]. Most commercial superabsorbent polymers are in the form of particles that retain more fluid when mechanically compressed. Increasing the particle size results in a large increase in the water absorption capacity but a decrease in the water absorption rate. However, if the particle size is considerably small, it will have more limitations in practical applications. For example, the pore space of the coating material should not be too large; otherwise, particles will leak out easily, and the particles, after absorbing water, will come into contact with the skin and other parts of the body, resulting in a poor experience of use [143]. In addition, the shape of the particles [144] has a significant impact on their absorption properties and mechanical strength; angular particles have a larger surface area and poorer mobility than spherical particles.

### 3.2. Superabsorbent Polymer Fibers

**Figure 8:**
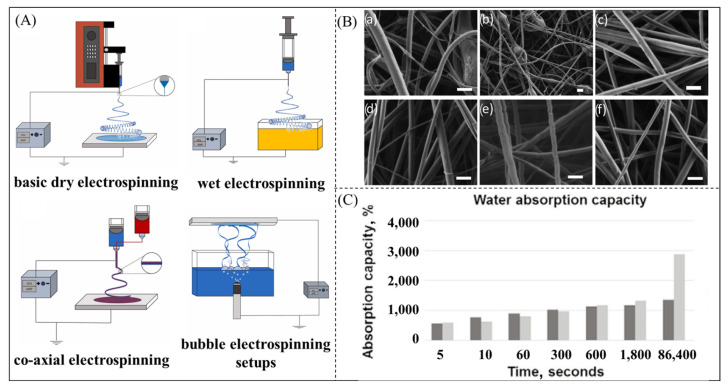
(**A**) Electrostatic spinning technique [147]. Copyright 2022, reproduced with permission of Elsevier. (**B**): (a) Branched fibers at high magnification; (b) branched fibers at low magnification; (c) pure straight-chain starch fibers; (d–f) fibers containing 28%, 50%, and 70% AM, respectively. Scale bar = 500 nm. [151]. Copyright 2019, reproduced with permission of Elsevier. (**C**) Comparison of the water uptake capacity of electrostatically spun PVP and PAA nanofibers [152]. Copyright 2021, reproduced with permission of Institutional National de Cercetare-Dezvoltare Pentru Texttile Pielarie.

Superabsorbent fibers are prepared by dry spinning [69], wet spinning [145], and electrostatic spinning [146], which can be combined with conventional spinning techniques (Figure 8A) to prepare nanoscale superabsorbent fibers [147,148,149]. Chen et al. [150] investigated the fractal dimension of the pore area of superabsorbent polymer fibers, and the results indicated that the larger the pore area, the stronger the adsorption capacity and the higher the water absorption rate. Vasilyev et al. [151] prepared electrostatically spun nanofibers using straight-chain starch and branched-chain starch (Figure 8B) and investigated the impact of polysaccharide types and proportions on the morphology and performance of fibers. The results revealed that the fiber morphology was relatively uniform. The fibers enriched with amylose exhibited higher strength, stiffness, and ductility, whereas those enriched with amylopectin were weaker and more brittle. Güler et al. [152] prepared polyvinylpyrrolidone (PVP) and PAA nanofibers by electrostatic spinning under optimal process parameters and analyzed and compared the water absorbency properties of the two types of fibers. The absorbency of the PVP nanofibers was higher than that of PAA; however, the PAA nanofibers had higher absorbency than the PAA nanofibers, but the PAA nanofibers had higher absorbency. This is mainly because PVP fibers have a finer diameter and higher porosity, with a larger surface area of nanofibers providing more water absorption sites, which is conducive to the penetration and adsorption of water, and therefore have a higher absorption rate than the PAA fibers. However, the PAA fibers contain a larger number of water-absorbing groups and therefore have a higher water absorption capacity.

The hydrophilic modification of fibers is currently the most commonly used method for producing superabsorbent fibers, in which normal fibers are physically or chemically treated to improve their water absorption [153,154]. Physical modifications involve blending normal fibers with water-absorbent fibers, adding water-absorbent powder to normal fibers, or coating fibers with a solution of superabsorbent resins to increase water absorption. Conversely, chemical treatment involves reacting normal fibers with reactive substances to obtain highly absorbent fibers.

### 3.3. Superabsorbent Polymer Gels

**Figure 9:**
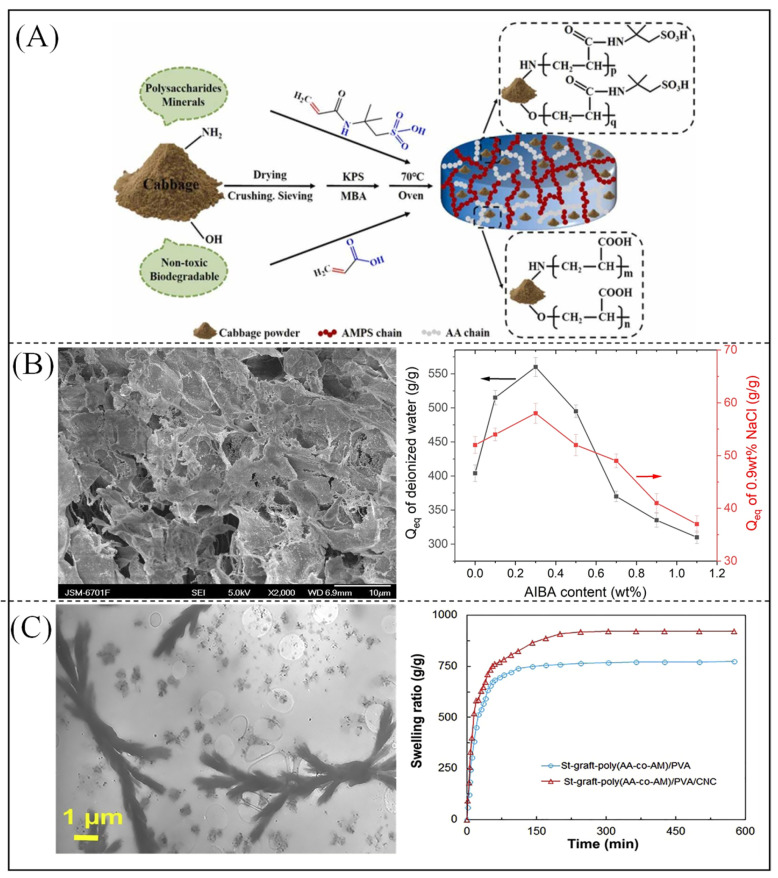
(**A**) Schematic of the synthesis process of highly absorbent polymer gel for kale [156]. Copyright 2021, reproduced with permission of Elsevier. (**B**) SEM of porous hydrogel and the effect of porogenic agent on water absorption [157]. Copyright 2023, reproduced with permission of Springer Nature. (**C**) TEM image and swelling rate of composite hydrogel material [158]. Copyright 2020, reproduced with permission of Elsevier.

Polymers undergo crosslinking to directly form hydrogels, which involves chemical reactions or physical interactions (e.g., ionic interactions and hydrogen bonding) [155]. As shown in Figure 9A, a superabsorbent gel with the water uptake of 1914 g/g has been synthesized using monomers of 2-acrylamido-2-methyl-1-propanesulfonic acid and AA [156].

In addition, studies have shown that gels prepared using different polymerization methods exhibit different water absorption properties. Zhai et al. [157] used a combination of foaming and back-surface crosslinking of a novel porogenic agent, 2,2′-azino bis(2-amidinopropane) dihydrochloride (AIBA), to prepare fast-swelling porous PAA/PVA high absorbency hydrogels with high pressurized brine absorption and investigated the impact of AIBA content on hydrogel water absorption (as shown in Figure 9B). Olad et al. [158] synthesized highly absorbent hydrogel composites based on starch-grafted PAA polyacrylamide/poly(vinyl alcohol) and cellulose using a radical water polymerization method, and the composite gels exhibited a higher equilibrium swelling rate than pure hydrogels (772.4 g/g) (921.8 g/g), as shown in Figure 9C.

Freeze-drying of hydrogels results in aerogels with ultrahigh porosity and ultralow thermal conductivity, enabling them to provide more water absorption space and channels, thereby increasing water absorption [159].

## 4. Applications of Superabsorbent Polymers

### 4.1. Industrial Applications

**Figure 10:**
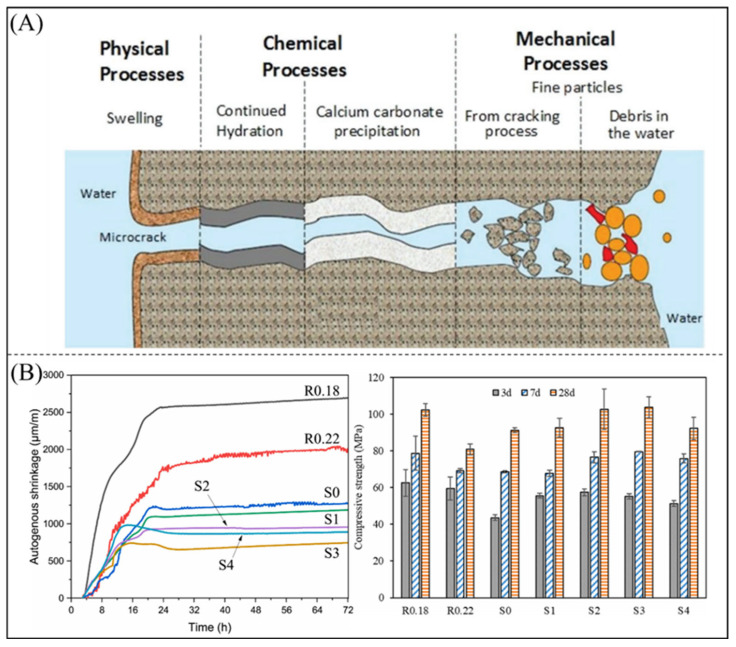
(**A**) Main mechanism of self-healing [160]. Copyright 2018, reproduced with permission of John Wiley and Sons. (**B**) Changes in self-shrinkage and compressive strength of cement pastes with different particle sizes of SAP powders [161]. Copyright 2023, reproduced with permission of John Wiley and Sons.

High-performance concrete inevitably cracks due to aging and degradation, and several studies have shown that the addition of superabsorbent polymers to cement can promote its “self-healing”. Figure 10A shows the self-healing mechanism of concrete which superabsorbent polymers assist in. On one hand, it continually hydrates the unhydrated cement particles. On the other hand, the Ca^2+^ present in the concrete matrix chemically reacts with the available CO_3_^2−^ in water or CO_2_ in the air, resulting in the formation of calcium carbonate, which deposits itself on the crack surface [160]. Superabsorbent polymers can also serve as internal curing agents, effectively mitigating the autogenous shrinkage of high-performance concrete, fostering cement hydration, and enhancing compressive strength. As shown in Figure 10B, Liu et al. [161] developed an ultrafine superabsorbent polymer powder and found that reducing the particle size of superabsorbent polymers resulted in reduced spacing in the cement paste. This is conducive to internal curing, but a diameter of less than 10 μm prevents the improvement of internal curing owing to agglomeration of superabsorbent polymer particles.

Rapid industrialization and a large population increase have caused serious water pollution, and research on wastewater treatment using superabsorbent polymer membranes has attracted increasing attention in recent decades. Feng et al. [162] prepared a superabsorbent polymer for adsorption of dyes, which possessed strong mechanical stability and excellent adsorption capacity. Zhang et al. [163] prepared superabsorbent hydrogel beads with sodium alginate and carboxymethyl chitosan with a maximum MB adsorption capacity of 2518 mg/g.

### 4.2. Agricultural Applications

**Figure 11:**
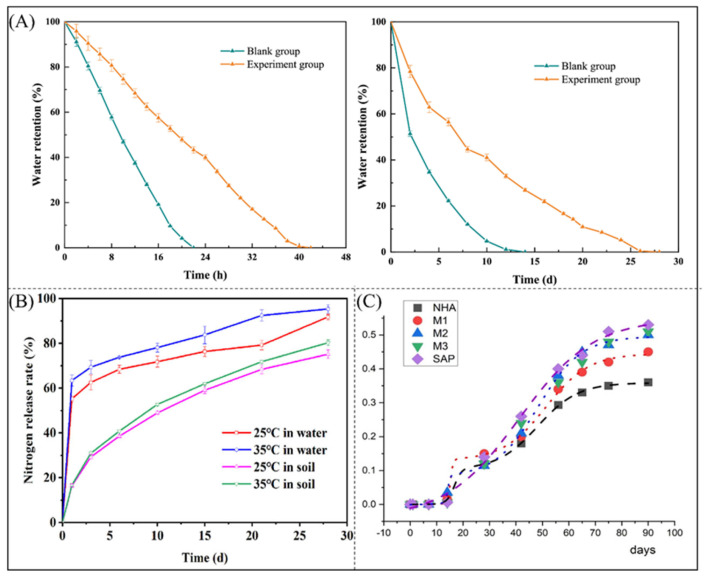
(**A**) Water retention time and water retention characteristics after mixed salt adsorption [164]. Copyright 2023, reproduced with permission of Elsevier (**B**) Nitrogen release characteristics [166]. Copyright 2023, reproduced with permission of Elsevier. (**C**) Nitrogen release curves [57]. Copyright 2023, reproduced with permission of John Wiley and Sons.

Superabsorbent polymers have also been used as water-saving materials and soil conditioners. The expansion and contraction of superabsorbent polymers during water cycling improve the porosity of clayey soils, thereby mitigating the adverse effects of drought conditions on plant growth. Zhang et al. [164] developed a superabsorbent polymer for use in saline soils, which showed excellent stability under high-salt conditions and extended water retention time up to 28 days. As shown in Figure 11A, the superabsorbent polymer extended the water retention time of the mixed saline solution by 20 h, and the soil with the superabsorbent polymer extended the water retention time to 28 days compared with the blank control. Ayoub et al. discovered that sodium alginate-based hydrogel mitigates the adverse effects of water scarcity on various growth parameters [165]. In addition to improving the soil water-use efficiency, superabsorbent polymers are used for the controlled release of fertilizers. Since nitrogen and phosphorus in fertilizers are highly soluble in water and tend to diffuse and volatilize, resulting in inadequate uptake by crops, loading fertilizers into superabsorbent polymers not only improves the efficiency of fertilizer use but also avoids unnecessary contamination. Insecticides or herbicides can also be loaded onto superabsorbent polymers for controlled release to avoid environmental pollution caused by the misuse of pesticides. A highly absorbent, water-retentive, and biodegradable superabsorbent material which can slowly release nitrogen fertilizers was prepared by copolymerization of carrageenan, AA, MBA, urea, and APS as raw materials [166], as shown in Figure 11B. Jancar et al. [57] optimized the nitrogen release profile by loading urea onto a superabsorbent starch polymer, as shown in Figure 11C.

### 4.3. Biomedical Applications

#### 4.3.1. Drug Delivery

**Figure 12:**
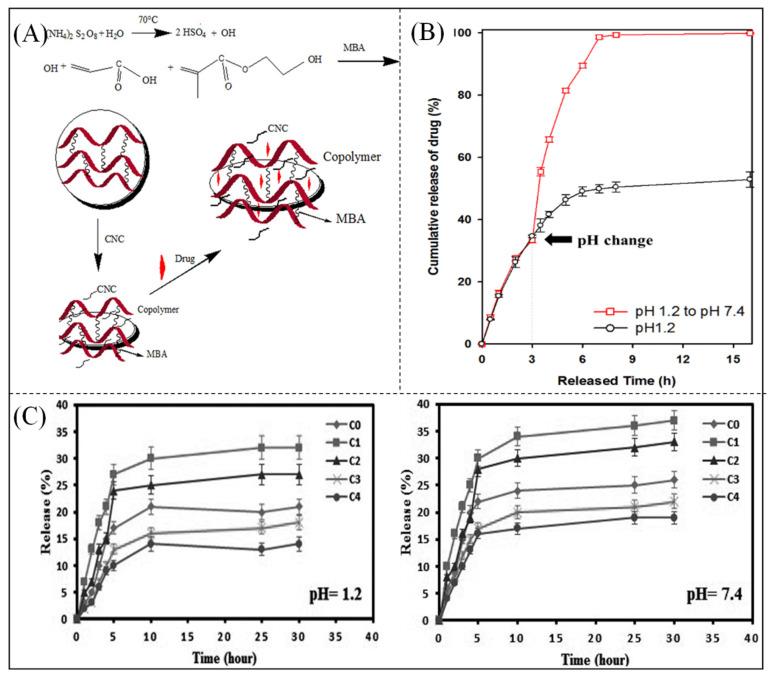
(**A**) Mechanism of superabsorbent hydrogel formation [168]. Copyright 2019, reproduced with permission of John Wiley and Sons. (**B**) Cumulative release of 5-FU from hydrogels at 37 °C [171]. Copyright 2021, reproduced with permission of MDPI. (**C**) Drug release behavior of gelatin-hydrogel nanocomposites [172]. Copyright 2018, reproduced with permission of John Wiley and Sons.

Because hydrogels are sensitive to environmental conditions, the controlled release of drugs can be achieved by loading them onto superabsorbent polymer hydrogels [31,167,168,169]. Hosseinzadeh et al. [170] prepared hydrogels based on AA and methacrylic acid 2-hydroxyethyl ester copolymers grafted with cellulose for controlled release of the drug ceftriaxone. The mechanism of hydrogel formation is illustrated in Figure 12A. Evidently, pH had a strong influence on the swelling behavior of the hydrogel, reaching a maximum swelling ratio of 92 g/g at a pH of 8. Figure 12B shows a graph of the in vitro release of 5-fluorouracil (5-FU) [171], which clearly shows the difference in release pattern according to pH, indicating that the prepared hydrogel exhibited pH-dependent swelling properties and that neutral pH showed a significant increase in the rate and amount of drug release under acidic conditions. Bakravi et al. [172] enhanced the mechanical properties of gelatin-based hydrogels with copper oxide nanoparticles and tested the drug delivery properties of the composites using cefadroxil as a model drug. Figure 12C shows the release profiles of model drugs under various pH conditions. At a pH of 7.4, more drug was released from the hydrogel nanocomposites than at a pH of 1.2 due to greater swelling. There is a direct relationship between drug release and swelling. When a hydrogel swells, it undergoes a transition from a dry to a swollen state, which greatly increases the mobility of drug molecules in the softened matrix and leads to drug release. Thus, the drug release mechanism was controlled by solubilization.

In addition, combining drugs with superabsorbent polymer solutions for electrostatic spinning and varying the polymer type, environmental parameters, equipment control parameters, and solution parameters can yield nanofibers with different diameters and morphologies, resulting in personalized drug release profiles and improved pharmacokinetic parameters [173,174,175].

#### 4.3.2. Wound Dressing

Electrostatically spun superabsorbent polymer nanofiber membranes have an extremely high specific surface area, which can provide more cell adhesion and growth sites [176,177]. Moreover, they can better mimic the size and structure of the protein collagen fibers present in the extracellular matrix of natural skin organoids, creating a microenvironment that promotes wound healing and skin regeneration [178,179] Superabsorbent nanofiber membranes prepared by electrostatic spinning have high porosities and small pore sizes, which can effectively block the invasion of external pathogens and provide good air and moisture permeability [180,181]. The nanofibers can also be loaded with various bioactive and therapeutic agents by electrostatic spinning to improve wound healing efficiency [182,183].

**Figure 13:**
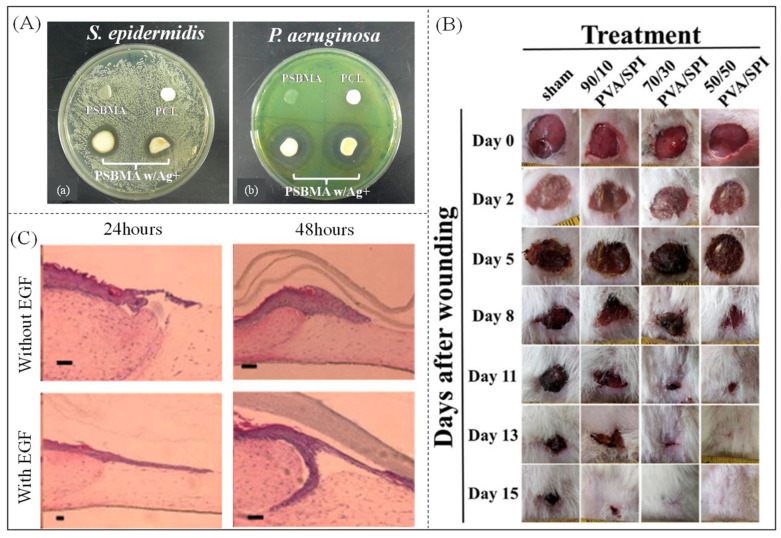
(**A**) Zone of inhibition of (a) *Staphylococcus epidermidis* and (b) *Pseudomonas aeruginosa* by fibrous membrane [184]. Copyright 2012, reproduced with permission of American Chemical Society. (**B**) Schematic of wound healing in mice [186]. Copyright 2012, reproduced with permission of American Chemical Society. (**C**) H&E staining of wounds after 24 h and 48 h. Scale bar = 500 nm. [176]. Copyright 2009, reproduced with permission of Elsevier.

Lalani et al. [184] prepared electrospun poly (sulfobetaine methacrylate) fibrous membranes for wound dressings using a three-step polymerization–electrostatic spinning–photo-crosslinking method. As shown in Figure 13A, the prepared nanofibrous membranes exhibited antibacterial activity against both *Staphylococcus epidermidis* and *Pseudomonas aeruginosa*; the surface of the prepared nanofibrous membranes was resistant to protein adsorption, and cells could not adhere to the surface. Electrospun fiber membranes have superior water absorption and repeated water absorption compared to hydrogel polymers and are highly resistant to protein adsorption, cell adhesion and proliferation, and bacterial adhesion. Other researchers have promoted wound healing by loading bioactive agents onto superabsorbent nanofiber membranes. Gaydhane et al. [185] prepared multilayered electrospun PVA/cellulose acetate nanofiber mats loaded with curcumin and honey for wound dressing. The multilayered PVA/CA nanofiber membrane could maximize the rapid absorption of wound fluid and maintain a wet state without deformation. The curcumin and honey encapsulated in the fibers could be released in a controlled manner to produce synergistic antioxidant, antibacterial, and anti-inflammatory effects to control wound development and promote healing. Gao et al. [182] developed dimethyl oxaloacetylglycine (DMOG) nanofiber wound dressings to inhibit hypoxia-induced wound growth and healing through its sustained release. DMOG inhibits the degradation of hypoxia-inducible factor 1α and subsequently improves diabetic wound regeneration by accelerating re-epithelialization, angiogenesis, and wound closure. Varshney et al. [186] used a physically crosslinked, highly absorbent poly(vinyl alcohol)/soybean isolate protein hydrogel as a dermal wound dressing and investigated the healing of excisional wounds in a rat model over 15 days, as shown in Figure 13B. The samples exhibited significantly less adhesion to the wound bed, and histological staining showed that the sample group exhibited wound re-epithelialization and collagen deposition in the dermis, suggesting that this hydrogel promoted wound healing. Schneider et al. [176] loaded epidermal growth factor (EGF) onto sericin protein nanofibrous membranes via electrostatic spinning, as shown in Figure 13C. Then, 24 and 48 h after wounding, the tissue was analyzed morphologically with hematoxylin and eosin, and wound closure was assessed by measuring the distance between the wound edges and the tip of the epithelial tongue as a percentage of wound closure. The wounds covered with fibrous membranes containing the growth factor EGF showed longer epithelial tongues at 24 h, and the results at 48 h were similar to those at 24 h, suggesting that EGF can be released from the nanofibrous membrane and consistently promote wound healing.

Superabsorbent polymer materials, such as wound dressings, can rapidly absorb wound fluid to keep the wound moist and its environment conducive to healing without causing secondary damage to the wound due to polymer dissolution. Such materials can also be loaded with a variety of drugs for antibacterial and anti-inflammatory purposes to prevent wound infection and promote rapid wound healing [187]. The wide use of superabsorbent polymer materials in wound dressings is mainly due to the following advantages: (1) high absorbency and ability to remain intact after dissolution equilibrium, (2) biocompatibility, (3) ability to create an osmotic gradient, (4) ability to form a gel, (5) ability to trap harmful ions, and (6) ability to absorb and trap fluids, bacteria, etc., in the dressing [188,189].

#### 4.3.3. Tissue Engineering

Scaffolds made of superabsorbent polymer materials are used to deliver drugs, genes, cells, or implants into the human body [190,191,192]. These materials should have good mechanical properties, and superabsorbent polymer hydrogels should not rupture when stretched under significant strain compared to conventional polymer hydrogels.

**Figure 14:**
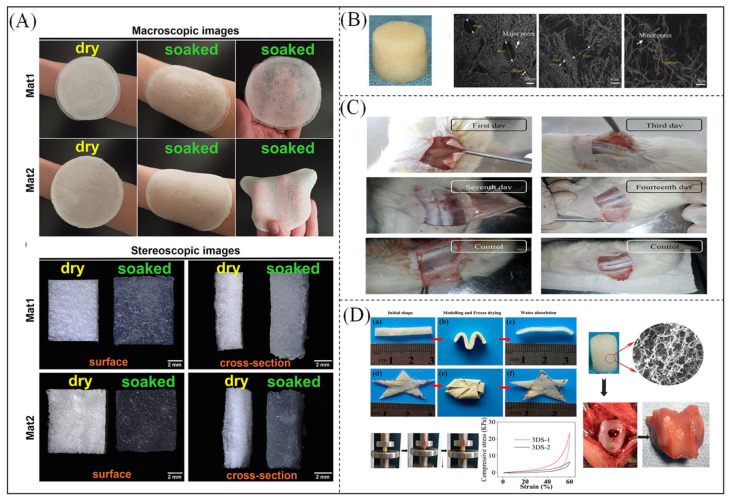
Images of gellan gum/agarose and gellan gum/chitosan biomaterials in their dry and wet states. (**A**) Mat1 (curdlan/agarose) and Mat2 (curdlan/chitosan) biomaterials in a dry and wet state: macroscopic and stereoscopic visualization [193]. Copyright 2021, reproduced with permission from Elsevier). (**B**) Digital camera images of 3D scaffolds and SEM images at different magnifications [194]. Copyright 2016, reproduced with permission of Elsevier. (**C**) Images of rat back wounds at 1, 3, 7, and 14 d of evaluation, respectively [197]. Copyright 2021, reproduced with permission from Elsevier). (**D**) Porous 3D scaffolds of electrospun gelatin/PLA nanofibers for cartilage tissue regeneration. Photographs showing the scaffold shape recovery after water absorption (3DS-2). (a–c) showed rectangular scaffold; (d–f) showed quinquangular scaffold. [198]. Copyright 2016, reproduced with permission from the American Chemical Society.

Wojcik et al. [193] prepared superabsorbent hybrid bioscaffolds from agarose and chitosan and obtained foamy microstructures with a high water absorption capacity by lyophilization. As shown in Figure 14A, the material has a spongy structure in a dry state, whereas wetting biomaterials have hydrocolloidal properties that produce a very smooth and soft gel that easily adheres to the wound bed. Chen et al. [194] used a combination of electrostatic spinning and freeze-drying techniques to prepare 3D nanofibrous scaffolds with a structure similar to that of collagen in the ECM, as shown in Figure 14B. In the scaffolds, obvious cellular structures such as circles, ovals, triangles, and other irregular shapes were observed. The pores with diameters of about 100–500 μm can be attributed to cryosolvent crystals formed during freeze-drying. Yang et al. [195] used maleic anhydride to graft glucose to improve its mechanical properties and electrospun it with gelatin to obtain photo-crosslinked composite fibers which have good biocompatibility and can be applied in tissue engineering. Sartore et al. [196] incorporated sodium polyacrylate particles as fillers into the PLA matrix to obtain superabsorbent composites. The addition of sodium polyacrylate, which swells in an aqueous environment and leaches out of the matrix to produce a very high porosity, improves the mechanical properties while maintaining its high water absorption capacity. Mahmoodzadeh et al. [197] synthesized a novel highly absorbent material by combining a silica aerogel and calcium chloride-modified CMC-Na and hydroxyl ethyl cellulose using both chemical and physical crosslinking methods, which can be used to produce highly absorbent materials by inducing the accumulation of erythrocytes and platelets at the bleeding site to form a fibrin network to control massive bleeding. As shown in Figure 14C, on Day 1, the polymer lost its normal state and turned yellow. By day 3, a large amount of gel was absorbed. On day 14, the gel structure degraded and was absorbed by the rats, except at the incision site, which was completely improved, with only a partially viscous state existing under the skin. The results showed that the novel highly absorbent material not only controlled massive bleeding at the wound site but also degraded directly in the organism. Chen et al. [198] prepared porous 3D scaffolds with gelatin and PLA and crosslinked them with hyaluronic acid to improve cartilage repair (Figure 14D). The in vivo cartilage regeneration of 3D scaffolds was investigated in rabbits using an articular cartilage injury model. These scaffolds exhibited excellent water absorption and cytocompatibility. In vivo studies have demonstrated that scaffolds can enhance cartilage repair.

In the biomedical field, when working with superabsorbent polymers, it is crucial to consider not only biocompatibility but also stability. However, many electrospun nanofiber scaffolds are less stable in aqueous environments because they swell and collapse into thin films upon contact with water. This results in a large reduction in the pore number and thickness, which hinders the spreading of cells and limits their use in biomedical applications [199]. In contrast to 2D nanofibrous scaffolds, 3D nanofibrous scaffolds are more similar to the in vivo environment in which cells survive while regulating their physiological responses to cell growth, migration, differentiation, and mechanistic remodeling [200]. Currently, the study of the in vivo degradation of superabsorbent polymers remains a hot topic.

## 5. Conclusions and Outlook

Superabsorbent polymers have superabsorption abilities and specific retention ability for water or other body fluids. Their main forms are particles, fibers, gels, and films, which are widely used in many fields. PAA polymers are the most common synthetic superabsorbent polymers. The initiator, crosslinking agent, and temperature during the synthesis process affect the water absorption properties of PAA polymers. As the degradation performance of polyacrylate is poor and prone to causing environmental pollution, inorganic additives are added to PAA polymers to increase their solubility and improve degradation performance. In addition, after the hydrolysis of polyacrylonitrile with an alkali, the hydrophobic group is transformed into an alkali metal carboxylic acid group that enhances the water-absorbing ability of PAN. Furthermore, the biodegradation performance of PVA can be enhanced after chemical modification. Cellulose, chitosan, starch, and alginate also have certain superabsorbent properties and can be modified by grafting to improve their water absorption capacities. Superabsorbent polymers can be used as additives in concrete materials to promote internal curing, as adsorbents for removing pollutants and toxic heavy metals from wastewater, as soil conditioners to reduce the adverse effects of drought on plant growth, and as corrosion inhibitors for underground cables, coal dewatering, and food preservation. Innovations in the design of functional materials have advanced the research on superabsorbents in biomedical applications. Studies have shown that superabsorbent polymers can be used as carriers for drug delivery, wound dressings, tissue-engineered scaffolds, etc.

The perspectives of superabsorbent polymers lie mainly in the following three aspects. The first one is the continuous synthesis of new types of superabsorbent polymers, and endowing them with better compatibility, easier processability, and greater absorbability. Among those polymers, superabsorbent hydrogel should be a brilliant star due to its broad potential biomedical applications [56,94,201]. The second one is to convert the superabsorbent polymers into a suitable format to take advantage of their desired functions. Among various formats, electrospun fibrous film is the most anticipated one. Over the past several decades, electrospinning has moved forward from the single-fluid process [202,203,204], to coaxial [205,206,207], triaxial [208,209], side-by-side [210], and combination(s) thereof [211]. The resultant multi-chamber fibrous structures would provide powerful support platforms for the new functional products. The third one is expanding new kinds of functional applications that can better people’s lives, such as the maintenance of home humidity, biomedical devices, and moisture removal for storage of food. Certainly, with further refinement and in-depth research, the range of applications of superabsorbent polymers will continue to expand.

## Figures and Tables

**Table 1:** Superabsorbent polymers and their applications.

Material	Molecular Weight (Monomer)	Physical Property	Water Absorbency (g/g)	Water Holding Rate	Application	Hydrophilic Group	Reference
Polyacrylic acid	72	Colorless/light yellow liquid	59	>90%	Horticulture, agriculture, drug delivery	–COOH	[39,40]
Polyacrylonitrile	53	White opaque powder	95	85%	Drug delivery, construction work, oil and water separation	–CH_3_NO,–COOR	[41,42,43]
Polyvinyl alcohol	44	Flocculent, granular, powdery white solid	74	93%	Cement, adsorption of alkali metals, cardiac tissue engineering	–COOH	[44,45,46,47]
Cellulose	162	Macromolecular polysaccharide	119	61%	Soil water retention conditioning	–COOH	[48,49,50]
Chitosan	161	White or off-white, semi-transparent, flaky or powdery solid	670	56%	Soil water retention, isolated lysozyme, wound dressings	–SO_3_H	[51,52,53]
Starch	162	White powdery solid	343	80%	Soil water retention, flexible batteries, controlled release fertilizers	–OH, –COOH	[54,55,56,57]
Protein	75–240	A macromolecule consisting of n peptide chains	860	30%	Hygiene products, hemostatic agents	–CO–NH	[58,59,60]
Amino acid	75–240	White crystals	590	55%	Viral diagnostic monitoring, wound dressing, drug delivery	–CO–NH	[61,62,63]
Alginate	176	White or light yellow powder	1700	70%	Dye adsorption, water-absorbent fabrics, concrete anti-cracking	–COONa,–OH	[64,65,66]

## Data Availability

No new data were created or analyzed in this study. Data sharing is not applicable to this article.

## References

[1] Buchholz F.L., Graham A.T. (1998). Modern Superabsorbent Polymer Technology.

[2] Fanta G.F., Burr R.C., Russell C.R., Rist C.E. (1966). Graft copolymers of starch. I. Copolymerization of gelatinized wheat starch with acrylonitrile. Fractionation of copolymer and effect of solvent on copolymer composition. J. Appl. Polym. Sci..

[3] Fanta G.F., Burr R.C., Russell C.R., Rist C.E. (1966). Graft copolymers of starch. II. Copolymerization of gelatinized wheat starch with acrylonitrile: Influence of reaction conditions on copolymer composition. J. Polym. Sci. Part B Polym. Lett..

[4] Fanta G.F., Burr R.C., Russell C.R., Rist C.E. (1967). Graft copolymers of starch. III. Copolymerization of gelatinized wheat starch with acrylonitrile. Influence of chain modifiers on copolymer composition. J. Appl. Polym. Sci..

[5] Athawale V.D., Lele V. (2001). Recent trends in hydrogels based on starch-graft-acrylic acid: A review. Starch.

[6] Zohuriaan-Mehr M.J., Kabiri K. (2008). Superabsorbent polymer materials: A review. Iran. Polym. J..

[7] Gooch J.W. (2011). Super absorbent fibers. Encyclopedic Dictionary of Polymers.

[8] Fanta G.F., Burr R.C., Doane W.M., Russell C.R. (1978). Absorbent polymers from starch and flour through graft polymerization of acrylonitrile and comonomer mixtures. Starch.

[9] Capezza A., Newson W.R., Olsson R.T., Hedenqvist M.S., Johansson E. (2019). Advances in the use of protein-based materials: Toward sustainable naturally sourced absorbent materials. ACS Sustain. Chem. Eng..

[10] Ma J., Li X., Bao Y. (2015). Advances in cellulose-based superabsorbent hydrogels. RSC Adv..

[11] Zhang J., Wang Q., Wang A. (2007). Synthesis and characterization of chitosan-g-poly(acrylic acid)/attapulgite superabsorbent composites. Carbohydr. Polym..

[12] Laftah W.A., Hashim S., Ibrahim A.N. (2011). Polymer hydrogels: A review. Polym. Plast. Technol. Eng..

[13] Zohuriaan-Mehr M.J., Omidian H., Doroudiani S., Kabiri K. (2010). Advances in non-hygienic applications of superabsorbent hydrogel materials. J. Mater. Sci..

[14] Cheng B., Pei B., Wang Z., Hu Q. (2017). Advances in chitosan-based superabsorbent hydrogels. RSC Adv..

[15] Zohuriaan-Mehr M.J., Pourjavadi A., Salimi H., Kurdtabar M. (2009). Protein- and homo poly(amino acid)-based hydrogels with super-swelling properties. Polym. Adv. Technol..

[16] Liu Q.X., Ding Z.R., Dong Z. (2012). Swelling behaviors of acrylic-based superabsorbent fibers. Adv. Mater. Res..

[17] Feng D., Bai B., Ding C., Wang H., Suo Y. (2014). Synthesis and Swelling Behaviors of Yeast-*g*-poly(acrylic acid) Superabsorbent Co-polymer. Ind. Eng. Chem. Res..

[18] Pourjavadi A., Zohuriaan-Mehr M.J., Ghasempoori S.N., Hossienzadeh H. (2007). Modified CMC. V. Synthesis and super-swelling behavior of hydrolyzed CMC-*g*-PAN hydrogel. J. Appl. Polym. Sci..

[19] Mucientes A.E., Santiago F., Delgado A.M. (2005). Effect of initial N,N′-methylene bisacrylamide concentration on the swelling behaviour of acrylic-based superabsorbent polymers. Pol. J. Chem..

[20] Fang S., Wang G., Xing R., Chen X., Liu S., Qin Y., Li K., Wang X., Li R., Li P. (2019). Synthesis of superabsorbent polymers based on chitosan derivative graft acrylic acid-co-acrylamide and its property testing. Int. J. Biol. Macromol..

[21] Utech S., Boccaccini A.R. (2016). A review of hydrogel-based composites for biomedical applications: Enhancement of hydrogel properties by addition of rigid inorganic fillers. J. Mater. Sci..

[22] Husain M.S.B., Gupta A., Alashwal B.Y., Sharma S. (2018). Synthesis of PVA/PVP based hydrogel for biomedical applications: A review. Energy Sources Part A Recover. Util. Environ. Eff..

[23] Shalla A.H., Yaseen Z., Bhat M.A., Rangreez T.A., Maswal M. (2019). Recent review for removal of metal ions by hydrogels. Sep. Sci. Technol..

[24] Atta A.M., Abdel-Rahman A.A.-H., El Aassy I.E., Ahmed F.Y., Hamza M.F. (2010). Adsorption properties of uranium (VI) ions on reactive crosslinked Acrylamidoxime and acrylic acid copolymer resins. J. Dispers. Sci. Technol..

[25] Zhang S., Chen H., Liu S., Guo J. (2017). Superabsorbent polymer with high swelling ratio, and temperature-sensitive and magnetic properties employed as an efficient dewatering medium of fine coal. Energy Fuels.

[26] Huang X., Jiang W., Zhou J., Yu D.-G., Liu H. (2022). The Applications of Ferulic-Acid-Loaded Fibrous Films for Fruit Preservation. Polymers.

[27] Mishra B., Upadhyay M., Reddy Adena S., Vasant B., Muthu M. (2017). Hydrogels: An introduction to a controlled drug delivery device, synthesis and application in drug delivery and tissue engineering. Austin J. Biomed. Eng..

[28] Wei Y., Zeng Q., Wang M., Huang J., Guo X., Wang L. (2019). Near-infrared light-responsive electrochemical protein imprinting biosensor based on a shape memory conducting hydrogel. Biosens. Bioelectron..

[29] Pontremoli C., Boffito M., Fiorilli S., Laurano R., Torchio A., Bari A., Tonda-Turo C., Ciardelli G., Vitale-Brovarone C. (2018). Hybrid injectable platforms for the in situ delivery of therapeutic ions from mesoporous glasses. Chem. Eng. J..

[30] Singh A., Vaishagya K., Verma R.K., Shukla R. (2019). Temperature/pH-triggered PNIPAM-based smart nanogel system loaded with anastrozole delivery for application in cancer chemotherapy. Aaps Pharmscitech.

[31] Lin X., Miao L., Wang X., Tian H. (2020). Design and evaluation of pH-responsive hydrogel for oral delivery of amifostine and study on its radioprotective effects. Colloids Surf. B Biointerfaces.

[32] Spicer C.D. (2020). Hydrogel scaffolds for tissue engineering: The importance of polymer choice. Polym. Chem..

[33] Mannarino M.M., Bassett M., Donahue D.T., Biggins J.F. (2020). Novel high-strength thromboresistant poly(vinyl alcohol)-based hydrogel for vascular access applications. J. Biomater. Sci. Polym. Ed..

[34] Wang M., Hou J., Yu D.-G., Li S., Zhu J., Chen Z. (2020). Electrospun tri-layer nanodepots for sustained release of acyclovir. J. Alloys Compd..

[35] Wang Y., Yu D.-G., Liu Y., Liu Y.-N. (2022). Progress of Electrospun Nanofibrous Carriers for Modifications to Drug Release Profiles. J. Funct. Biomater..

[36] Du J., Gao S., Shi P., Fan J., Xu Q., Min Y. (2020). Three-dimensional carbonaceous for potassium ion batteries anode to boost rate and cycle life performance. J. Power Sources.

[37] Buckingham M.A., Zhang S., Liu Y., Chen J., Marken F., Aldous L. (2021). Thermogalvanic and thermocapacitive behavior of superabsorbent hydrogels for combined low-temperature thermal energy conversion and harvesting. ACS Appl. Energy Mater..

[38] Yang Y., Wang H., Liu W., Shi J., Dong G., Zhang H., Li D., Lu G. (2019). Polymer salt-derived carbon-based nanomaterials for high-performance hybrid Li-ion capacitors. J. Mater. Sci..

[39] Mendoza D.J., Ayurini M., Raghuwanshi V.S., Simon G.P., Hooper J.F., Garnier G. (2023). Synthesis of superabsorbent polyacrylic acid-grafted cellulose nanofibers via silver-promoted decarboxylative radical polymerization. Macromolecules.

[40] Zhang Y., Wei J., Zhang X., Gao P. (2023). Fabrication and swelling properties of a novel superabsorbent composite derived from waste coal gangue. Polym. Polym. Compos..

[41] Sadeghi M., Gudarzi A., Safari S., Shahsavari H., Sadeghi H. (2013). Synthesis of superabsorbent hydrogels consisted of pectin and poly(methacrylonitrile) for drug delivery systems. Asian J. Chem..

[42] de Oliveira M.R., Gonçalves E.P. (2022). Addition of polyacrylonitrile superabsorbent polymer in cement mixture with variation in the amount of water. Rev. UniVap..

[43] Ge J., Jia Y., Cheng C., Sun K., Peng Y., Tu Y., Qiang Y., Hua Z., Zheng Z., Ye X. (2021). Polydimethylsiloxane-functionalized polyacrylonitrile nanofibrous aerogels for efficient oil absorption and oil/water separation. J. Appl. Polym. Sci..

[44] Ma C., Fan F., Chen M., Li S., Chen Y., Pan Z., Liu R. (2022). Preparation of a novel superabsorbent fiber–cement composite and evaluation of its self-healing performance. Cem. Concr. Compos..

[45] Sun J., Sun G., Zhao X., Liu X., Zhao H., Xu C., Yan L., Jiang X., Cui Y. (2021). Ultrafast and efficient removal of Pb(II) from acidic aqueous solution using a novel polyvinyl alcohol superabsorbent. Chemosphere.

[46] Kadhim S.A., Hameed A.M., Rasheed R.T. (2022). Synthesis and study of magnesium complexes derived from polyacrylate and polyvinyl alcohol and their applications as superabsorbent polymers. J. Mech. Behav. Mater..

[47] Sedighim S., Chen Y., Xu C., Mohindra R., Liu H., Agrawal D.K., Thankam F.G. (2023). Carboxymethyl cellulose–alginate interpenetrating hydroxy ethyl methacrylate crosslinked polyvinyl alcohol reinforced hybrid hydrogel templates with improved biological performance for cardiac tissue engineering. Biotechnol. Bioeng..

[48] Guo Y., Guo R., Shi X., Lian S., Zhou Q., Chen Y., Liu W., Li W. (2022). Synthesis of cellulose-based superabsorbent hydrogel with high salt tolerance for soil conditioning. Int. J. Biol. Macromol..

[49] Tian H., Cheng S., Zhen J., Lei Z. (2023). Superabsorbent polymer with excellent water/salt absorbency and water retention, and fast swelling properties for preventing soil water evaporation. J. Polym. Environ..

[50] Gao L., Luo H., Wang Q., Hu G., Xiong Y. (2021). Synergistic Effect of Hydrogen Bonds and Chemical Bonds to Construct a Starch-Based Water-Absorbing/Retaining Hydrogel Composite Reinforced with cellulose and poly(ethylene glycol). ACS Omega.

[51] Jayanudin, Lestari R.S.D., Barleany D.R., Pitaloka A.B., Yulvianti M., Prasetyo D., Anggoro D.V., Ruhiatna A. (2022). Chitosan-Graft-Poly(acrylic acid) Superabsorbent’s Water Holding in Sandy Soils and Its Application in Agriculture. Polymers.

[52] Akkaya R., Akkaya B., Çakıcı G.T. (2023). Chitosan–poly(acrylamide-co-maleic acid) composite synthesis, characterization, and investigation of protein adsorption behavior. Polym. Bull..

[53] Barleany D.R., Heriyanto H., Alwan H., Kurniawati V., Muyassaroh A., Erizal E. (2022). Effect of starch and chitosan addition on swelling properties of neutralized poly(acrylic acid)-based superabsorbent hydrogels prepared by using γ-irradiation technique. At. Indones..

[54] Yao Y., Shen Y., Hu C., Wu H. (2023). Superabsorbent fabric: In situ polymerisation of macroporous starch–sodium alginate–polyacrylate hydrogel on fibre surface. Cellulose.

[55] Xiong H., Peng H., Ye X., Kong Y., Wang N., Yang F., Meni B.-H., Lei Z. (2022). High salt tolerance hydrogel prepared of hydroxyethyl starch and its ability to increase soil water holding capacity and decrease water evaporation. Soil Tillage Res..

[56] Fan X., Zhang R., Sui S., Liu X., Liu J., Shi C., Zhao N., Zhong C., Hu W. (2023). Starch-Based superabsorbent hydrogel with high electrolyte retention capability and synergistic interface engineering for long-lifespan flexible zinc−air batteries. Angew. Chem. Int. Ed..

[57] Jancar J., Skarpa P., Mohsen-Latiff A. (2023). Fertilizer technology based on optimized nitrogen release from urea-loaded natural superabsorbent carriers. Soil Use Manag..

[58] Álvarez-Castillo E., Bengoechea C., Felix M., Guerrero A. (2021). Freeze-Drying versus Heat-Drying: Effect on Protein-Based Superabsorbent Material. Processes.

[59] Zhang W., Guo L., Liu Q., Yang M., Chen J., Lei Z. (2022). Preparation and properties of a biodegradability superabsorbent composite based on flax cake protein-g-poly (acrylic acid)/kaolinite. J. Appl. Polym. Sci..

[60] Jahan N., Mahbub S.I., Lee B.-T., Bae S.H. (2023). In Vivo and in vitro investigation of a novel gelatin/sodium polyacrylate composite hemostatic sponge for topical bleeding. J. Funct. Biomater..

[61] Chen W., Mei E., Xie X. (2022). Virus stabilization with enhanced porous superabsorbent polymer (PSAP) beads for diagnostics and surveillance. ACS ES&T Water.

[62] Meng H., Zhang X., Sun S., Tan T., Cao H. (2016). Preparation of γ-aminopropyltriethoxysilane cross-linked poly(aspartic acid) superabsorbent hydrogels without organic solvent. J. Biomater. Sci. Polym. Ed..

[63] Vakili M.R., Rahneshin N. (2013). Synthesis and characterization of novel stimuli-responsive hydrogels based on starch and L-aspartic acid. Carbohydr. Polym..

[64] Agnihotri S., Singhal R. (2019). Effect of sodium alginate content in acrylic acid/sodium humate/sodium alginate superabsorbent hydrogel on removal capacity of MB and CV dye by adsorption. J. Polym. Environ..

[65] Snoeck D., Moerkerke B., Mignon A., De Belie N. (2020). In-Situ crosslinking of superabsorbent polymers as external curing layer compared to internal curing to mitigate plastic shrinkage. Constr. Build. Mater..

[66] Nita L.E., Chiriac A.P., Ghilan A., Rusu A.G., Tudorachi N., Timpu D. (2021). Alginate enriched with phytic acid for hydrogels preparation. Int. J. Biol. Macromol..

[67] Yarimkaya S., Basan H. (2007). Synthesis and Swelling behavior of acrylate-based hydrogels. J. Macromol. Sci. Part A.

[68] Tang Q., Wu J., Lin J., Li Q., Fan S. (2008). Two-step synthesis of polyacrylamide/polyacrylate interpenetrating network hydrogels and its swelling/deswelling properties. J. Mater. Sci..

[69] Hu X.Y., Xiao C.F. (2005). Study on superabsorbent polyacrylonitrile-based fibre. Indian J. Fibre Text. Res..

[70] Bary E.M.A., Fekri A., Soliman Y.A., Harmal A.N. (2019). Manufacturing of superabsorbent membranes of PVA and rice husk fibres reinforced with nanosilica for agricultural and horticultural applications. Int. J. Environ. Stud..

[71] Ballard N., Asua J.M. (2018). Radical polymerization of acrylic monomers: An overview. Prog. Polym. Sci..

[72] Sharma K., Kumar V., Kaith B., Kumar V., Som S., Kalia S., Swart H. (2015). Synthesis, characterization and water retention study of biodegradable Gum ghatti-poly(acrylic acid–aniline) hydrogels. Polym. Degrad. Stab..

[73] Athawale V.D., Lele V. (2000). Factors influencing absorbent properties of saponified starch-g-(acrylic acid-co-acrylamide). J. Appl. Polym. Sci..

[74] Miyajima T., Matsubara Y., Komatsu H., Miyamoto M., Suzuki K. (2020). Development of a superabsorbent polymer using iodine transfer polymerization. Polym. J..

[75] Baloch F.E., Afzali D., Fathirad F. (2021). Design of acrylic acid/nanoclay grafted polysaccharide hydrogels as superabsorbent for controlled release of chlorpyrifos. Appl. Clay Sci..

[76] Raju K.M., Raju M.P., Mohan Y.M. (2002). Synthesis and water absorbency of crosslinked superabsorbent polymers. J. Appl. Polym. Sci..

[77] Ma S., Liu M., Chen Z. (2004). Preparation and properties of a salt-resistant superabsorbent polymer. J. Appl. Polym. Sci..

[78] Singh J., Dhaliwal A. (2018). Synthesis, characterization and swelling behavior of silver nanoparticles containing superabsorbent based on grafted copolymer of polyacrylic acid/Guar gum. Vacuum.

[79] Kwon Y.-R., Kim J.-S., Kim D.-H. (2021). Effective enhancement of water absorbency of itaconic acid based-superabsorbent polymer via tunable surface—Crosslinking. Polymers.

[80] Kwon Y.R., Lim S.H., Kim H.C., Kim J.S., Chang Y.W., Choi J., Kim D.H. (2021). Superabsorbent polymer with improved permeability and absorption rate using hollow glass microspheres. J. Polym. Sci..

[81] Stahl J.D., Cameron M.D., Haselbach J., Aust S.D. (2000). Biodegradation of superabsorbent polymers in soil. Environ. Sci. Pollut. Res..

[82] Hebeish A., Hashem M., El-Hady M.A., Sharaf S. (2013). Development of CMC hydrogels loaded with silver nano-particles for medical applications. Carbohydr. Polym..

[83] Anirudhan T.S., Suchithra P.S., Senan P., Tharun A.R. (2012). Kinetic and Equilibrium Profiles of Adsorptive Recovery of thorium(IV) from Aqueous Solutions Using poly(methacrylic acid) Grafted cellulose/bentonite Superabsorbent Composite. Ind. Eng. Chem. Res..

[84] Liu Y., Zhu Y., Wang Y., Mu B., Wang X., Wang A. (2022). Eco-friendly superabsorbent composites based on calcined semicoke and polydimethylourea phosphate: Synthesis, swelling behavior, degradability and their impact on cabbage growth. Colloids Surfaces A Physicochem. Eng. Asp..

[85] Soleimani F., Sadeghi M., Shasevari H., Soleimani A., Sadeghi H. (2013). Effective parameters onto swelling capacity of biosuperabsorbent based on acrylonitrile-sucrose. Asian J. Chem..

[86] Wang K.Y., Cen R.F., Shu W.W. (2015). Preparation and performance of super-absorbent resin using polyacrylonitrile fiber wastes. Adv. Mater. Res..

[87] Trivedi J., Chourasia A. (2023). Sodium salt of partially carboxymethylated sodium alginate-g-poly(acrylonitrile): I. Photo-induced synthesis, characterization, and alkaline hydrolysis. Gels.

[88] Bhattacharya S.S., Mishra A., Pal D., Ghosh A.K., Ghosh A., Banerjee S., Sen K.K. (2012). Synthesis and Characterization of poly(acrylic acid)/poly(vinyl alcohol)-xanthan gum Interpenetrating Network (IPN) Superabsorbent Polymeric Composites. Polym.-Plast. Technol. Eng..

[89] Oprea M., Voicu S.I. (2020). Recent advances in composites based on cellulose derivatives for biomedical applications. Carbohydr. Polym..

[90] Chen Y., Zhang Y., Wang F., Meng W., Yang X., Li P., Jiang J., Tan H., Zheng Y. (2016). Preparation of porous carboxymethyl chitosan grafted poly (acrylic acid) superabsorbent by solvent precipitation and its application as a hemostatic wound dressing. Mater. Sci. Eng. C Mater. Biol. Appl..

[91] Dragan E.S., Apopei D.F. (2011). Synthesis and swelling behavior of pH-sensitive semi-interpenetrating polymer network composite hydrogels based on native and modified potatoes starch as potential sorbent for cationic dyes. Chem. Eng. J..

[92] Sharma S., Dua A., Malik A. (2014). Polyaspartic acid based superabsorbent polymers. Eur. Polym. J..

[93] Zhao Y., Kang J., Tan T. (2006). Salt-, pH- and temperature-responsive semi-interpenetrating polymer network hydrogel based on poly(aspartic acid) and poly(acrylic acid). Polymer.

[94] Zhang Z., Abidi N., Lucia L., Chabi S., Denny C.T., Parajuli P., Rumi S.S. (2023). Cellulose/nanocellulose superabsorbent hydrogels as a sustainable platform for materials applications: A mini-review and perspective. Carbohydr. Polym..

[95] Demitri C., Del Sole R., Scalera F., Sannino A., Vasapollo G., Maffezzoli A., Ambrosio L., Nicolais L. (2008). Novel superabsorbent cellulose-based hydrogels crosslinked with citric acid. J. Appl. Polym. Sci..

[96] Hazmi A.T., Ahmad F.B., Akmal M.M., Ralib A.A.M., Ali F.B. (2023). Fungal chitosan for potential application in piezoelectric energy harvesting: Review on experimental procedure of chitosan extraction. Alex. Eng. J..

[97] Jolayemi O.L., Malik A.H., Ekblad T., Fredlund K., Olsson M.E., Johansson E. (2022). Protein-based biostimulants to enhance plant growth—State-of-the-art and future direction with sugar beet as an example. Agronomy.

[98] Sajilata M., Singhal R.S., Kulkarni P.R. (2006). Resistant starch–A review. Compr. Rev. Food Sci. Food Saf..

[99] Tester R.F., Karkalas J., Qi X. (2004). Starch—Composition, fine structure and architecture. J. Cereal Sci..

[100] Primarini D., Ohta Y. (2000). Some Enzyme Properties of Raw Starch Digesting Amylases from *Streptomyces* sp. No. 4. Starch.

[101] Harish Prashanth K.V., Tharanathan R.N. (2007). Chitin/chitosan: Modifications and their unlimited application potential—An overview. Trends Food Sci. Technol..

[102] Tang W.J., Fernandez J.G., Sohn J.J., Amemiya C.T. (2015). Chitin is endogenously produced in vertebrates. Curr. Biol..

[103] Chen Y., Liu Y.-F., Tan H.-M., Jiang J.-X. (2009). Synthesis and characterization of a novel superabsorbent polymer of N,O-carboxymethyl chitosan graft copolymerized with vinyl monomers. Carbohydr. Polym..

[104] Kean T., Thanou M., Williams P. (2011). Chitin and chitosan: Sources, production and medical applications. Renewable Resources for Functional Polymers and Biomaterials.

[105] Dash M., Chiellini F., Ottenbrite R.M., Chiellini E. (2011). Chitosan—A versatile semi-synthetic polymer in biomedical applications. Prog. Polym. Sci..

[106] Ma Z., Li Q., Yue Q., Gao B., Xu X., Zhong Q. (2011). Synthesis and characterization of a novel super-absorbent based on wheat straw. Bioresour. Technol..

[107] Jia Z., Shen D., Xu W. (2001). Synthesis and antibacterial activities of quaternary ammonium salt of chitosan. Carbohydr. Res..

[108] Johansson E., Malik A.H., Hussain A., Rasheed F., Newson W.R., Plivelic T., Hedenqvist M.S., Gällstedt M., Kuktaite R. (2013). Wheat gluten polymer structures: The impact of genotype, environment, and processing on their functionality in various applications. Cereal Chem..

[109] Wang Y., Li X., Zhang Y., Wang L., Yang Z. (2019). A supramolecular hydrogel to boost the production of antibodies for phosphorylated proteins. Chem. Commun..

[110] Jonker A.M., Löwik D.W.P.M., van Hest J.C.M. (2012). Peptide- and protein-based hydrogels. Chem. Mater..

[111] Min S.K., Kim J.H., Chung D.J. (2001). Swelling behavior of biodegradable crosslinked gel based on poly(aspartic acid) and PEG-diepoxide. Korea Polym. J..

[112] Kunioka M. (2004). Biodegradable water absorbent synthesized from Bacterial poly(amino acid)s. Macromol. Biosci..

[113] Lee K.Y., Mooney D.J. (2012). Alginate: Properties and biomedical applications. Prog. Polym. Sci..

[114] Qin Y. (2008). The gel swelling properties of alginate fibers and their applications in wound management. Polym. Adv. Technol..

[115] Manjula B., Varaprasad K., Sadiku R., Raju K.M. (2013). Preparation and characterization of sodium alginate–based hydrogels and their in vitro release studies. Adv. Polym. Technol..

[116] Hua S., Wang A. (2009). Synthesis, characterization and swelling behaviors of sodium alginate-g-poly(acrylic acid)/sodium humate superabsorbent. Carbohydr. Polym..

[117] Phang Y.-N., Chee S.-Y., Lee C.-O., Teh Y.-L. (2011). Thermal and microbial degradation of alginate-based superabsorbent polymer. Polym. Degrad. Stab..

[118] Sang Y., Zhao J.R. (2015). Reduction of water absorption capacity of cellulose fibres for its application in cementitious materials. J. Compos. Mater..

[119] Meimoun J., Wiatz V., Saint-Loup R., Parcq J., Favrelle A., Bonnet F., Zinck P. (2018). Modification of starch by graft copolymerization. Starch.

[120] Sawut A., Yimit M., Sun W., Nurulla I. (2014). Photopolymerisation and characterization of maleylatedcellulose-g-poly(acrylic acid) superabsorbent polymer. Carbohydr. Polym..

[121] Narayanan A., Dhamodharan R. (2015). Super water-absorbing new material from chitosan, EDTA and urea. Carbohydr. Polym..

[122] Athawale V., Lele V. (1998). Graft copolymerization onto starch. II. Grafting of acrylic acid and preparation of it’s hydrogels. Carbohydr. Polym..

[123] Qiao D., Liu H., Yu L., Bao X., Simon G.P., Petinakis E., Chen L. (2016). Preparation and characterization of slow-release fertilizer encapsulated by starch-based superabsorbent polymer. Carbohydr. Polym..

[124] Narmani A., Jafari S.M. (2021). Chitosan-based nanodelivery systems for cancer therapy: Recent advances. Carbohydr. Polym..

[125] Nge T.T., Hori N., Takemura A., Ono H. (2004). Swelling behavior of chitosan/poly(acrylic acid) complex. J. Appl. Polym. Sci..

[126] Wang X., Lou T., Zhao W., Song G. (2016). Preparation of pure chitosan film using ternary solvents and its super absorbency. Carbohydr. Polym..

[127] Raju M.P., Raju K.M. (2001). Design and synthesis of superabsorbent polymers. J. Appl. Polym. Sci..

[128] Tang H., Chen H., Duan B., Lu A., Zhang L. (2014). Swelling behaviors of superabsorbent chitin/carboxymethylcellulose hydrogels. J. Mater. Sci..

[129] Hanani Z.N., Roos Y., Kerry J. (2014). Use and application of gelatin as potential biodegradable packaging materials for food products. Int. J. Biol. Macromol..

[130] Daniele M.A., Adams A.A., Naciri J., North S.H., Ligler F.S. (2014). Interpenetrating networks based on gelatin methacrylamide and PEG formed using concurrent thiol click chemistries for hydrogel tissue engineering scaffolds. Biomaterials.

[131] Bagheri Marandi G., Beheshti Rouzbahani G., Kurdtabar M. (2014). Synthesis and swelling behavior of gelatin-based hydrogel nanocomposites. J. Appl. Chem. Res..

[132] Rathna G.V.N. (2004). Hydrogels of modified ethylenediaminetetraacetic dianhydride gelatin conjugated with poly(ethylene glycol) dialdehyde as a drug-release matrix. J. Appl. Polym. Sci..

[133] Pourjavadi A., Salimi H. (2008). New Protein-based hydrogel with superabsorbing properties: Effect of monomer ratio on swelling behavior and kinetics. Ind. Eng. Chem. Res..

[134] Song W., Zhang Y., Tran C.H., Choi H.K., Yu D.-G., Kim I. (2023). Porous organic polymers with defined morphologies: Synthesis, assembly, and emerging applications. Prog. Polym. Sci..

[135] Zhou J., Yi T., Zhang Z., Yu D.-G., Liu P., Wang L., Zhu Y. (2023). Electrospun Janus core (ethyl cellulose//polyethylene oxide) @ shell (hydroxypropyl methyl cellulose acetate succinate) hybrids for an enhanced colon-targeted prolonged drug absorbance. Adv. Compos. Hybrid Mater..

[136] Yu D.-G., Zhou J. (2023). How can Electrospinning Further Service Well for Pharmaceutical Researches?. J. Pharm. Sci..

[137] Fujita S., Tazawa T., Kono H. (2022). Preparation and enzyme degradability of spherical and water-absorbent gels from sodium carboxymethyl cellulose. Gels.

[138] Maijan P., Chantarak S. (2020). Synthesis and characterization of highly durable and reusable superabsorbent core–shell particles. Polym. Eng. Sci..

[139] Yang F., Zhang M., Yang H., Yan W., Jiang F. (2017). Effect of aggregate size on liquid absorption characteristics of konjac glucomannan superabsorbent. J. Appl. Polym. Sci..

[140] Sand A., Shin N.-J., Nam H.-G., Kwark Y.-J. (2021). Effects of Reaction Parameters on water Absorption of poly(itaconic acid) Superabsorbent Particles Synthesized by Inverse Suspension Polymerization. Fibers Polym..

[141] Soly S.J., Nosrati A., Skinner W., Addai-Mensah J. (2019). Superabsorbent-mediated dewaterability of fine hydrophobic sulphide mineral slurries. Sep. Sci. Technol..

[142] Fernández P., Kraemer F.B., Sabatté L., Guiroy J., Boem F.G. (2022). Superabsorbent polyacrylamide Effects on Hydrophysical Soil Properties and Plant Biomass in a Sandy Loam soil. Commun. Soil Sci. Plant Anal..

[143] Lee K.M., Min J.H., Oh S., Lee H., Koh W.-G. (2020). Preparation and characterization of superabsorbent polymers (SAPs) surface-crosslinked with polycations. React. Funct. Polym..

[144] Ramazani-Harandi M., Zohuriaan-Mehr M., Yousefi A., Ershad-Langroudi A., Kabiri K. (2006). Rheological determination of the swollen gel strength of superabsorbent polymer hydrogels. Polym. Test..

[145] Kim Y.-J., Yoon K.-J., Ko S.-W. (2000). Preparation and properties of alginate superabsorbent filament fibers crosslinked with glutaraldehyde. J. Appl. Polym. Sci..

[146] Petroudy S.R.D., Kahagh S.A., Vatankhah E. (2021). Environmentally friendly superabsorbent fibers based on electrospun cellulose nanofibers extracted from wheat straw. Carbohydr. Polym..

[147] Angel N., Li S., Yan F., Kong L. (2022). Recent advances in electrospinning of nanofibers from bio-based carbohydrate polymers and their applications. Trends Food Sci. Technol..

[148] Yu D.-G., Zhao P. (2022). The Key Elements for Biomolecules to Biomaterials and to Bioapplications. Biomolecules.

[149] Song W., Tang Y., Qian C., Kim B.J., Liao Y., Yu D.-G. (2023). Electrospinning spinneret: A bridge between the visible world and the invisible nanostructures. Innovation.

[150] Chen F.M., Zhu X.Z., Yang H.X. (2013). Effect of the pore fractal dimensions on absorption ability in superabsorbent fibers. Appl. Mech. Mater..

[151] Vasilyev G., Vilensky R., Zussman E. (2019). The ternary system amylose-amylopectin-formic acid as precursor for electrospun fibers with tunable mechanical properties. Carbohydr. Polym..

[152] Güler B., Çallioğlu F.C. (2021). Comparative analysis of superabsorbent properties of PVP and PAA nanofibres. Ind. Textila.

[153] Liu W., Lin H., Wang J., Han Q., Liu F. (2021). Polytetrafluoroethylene (PTFE) hollow fibers modified by hydrophilic crosslinking network (HCN) for robust resistance to fouling and harsh chemical cleaning. J. Membr. Sci..

[154] Jia E., Mou H., Liu Z., Wang J., Zeng L., Yang X., Liu P. (2020). Surface Hydrophilic Modification of Polypropylene Fibers and Their Application in Fiber-Reinforced Cement-Based Materials. J. Macromol. Sci. Part B Phys..

[155] Lu L., Yuan S., Wang J., Shen Y., Deng S., Xie L., Yang Q. (2018). The formation mechanism of hydrogels. Curr. Stem Cell Res. Ther..

[156] Zhang W., Liu Q., Guo L., Wang P., Liu S., Chen J., Lei Z. (2021). White Cabbage (*Brassica oleracea* L.) waste, as biowaste for the preparation of novel superabsorbent polymer gel. J. Environ. Chem. Eng..

[157] Zhai N., Wang B. (2023). Preparation of fast-swelling porous superabsorbent hydrogels with high saline water absorbency under pressure by foaming and post surface crosslinking. Sci. Rep..

[158] Olad A., Doustdar F., Gharekhani H. (2020). Fabrication and characterization of a starch-based superabsorbent hydrogel composite reinforced with cellulose nanocrystals from potato peel waste. Colloids Surfaces A Physicochem. Eng. Asp..

[159] Abdul Khalil H.P.S., Adnan A., Yahya E.B., Olaiya N., Safrida S., Hossain M.S., Balakrishnan V., Gopakumar D.A., Abdullah C., Oyekanmi A. (2020). A review on plant cellulose nanofibre-based aerogels for biomedical applications. Polymers.

[160] De Belie N., Gruyaert E., Al-Tabbaa A., Antonaci P., Baera C., Bajare D., Darquennes A., Davies R., Ferrara L., Jefferson T. (2018). A review of self-healing concrete for damage management of structures. Adv. Mater. Interfaces.

[161] Liu J., Wang M., Liu N., Teng L., Wang Y., Chen Z., Shi C. (2023). Development of ultra-fine SAP powder for lower-shrinkage and higher-strength cement pastes made with ultra-low water-to-binder ratio. Compos. Part B Eng..

[162] Feng D., Bai B., Wang H., Suo Y. (2018). Novel fabrication of PAA/PVA/Yeast superabsorbent with interpenetrating polymer network for pH-dependent selective adsorption of dyes. J. Polym. Environ..

[163] Zhang Z., Abidi N., Lucia L. (2023). Smart superabsorbent alginate/carboxymethyl chitosan composite hydrogel beads as efficient biosorbents for methylene blue dye removal. J. Mater. Sci. Technol..

[164] Zhang C., Meza J.V.G., Zhou K., Liu J., Song S., Zhang M., Meng D., Chen J., Xia L., Xiheng H. (2023). Superabsorbent polymer used for saline-alkali soil water retention. J. Taiwan Inst. Chem. Eng..

[165] El Idrissi A., Dardari O., Metomo F.N.N.N., Essamlali Y., Akil A., Amadine O., Aboulhrouz S., Zahouily M. (2023). Effect of sodium alginate-based superabsorbent hydrogel on tomato growth under different water deficit conditions. Int. J. Biol. Macromol..

[166] Li J., Zhu Y., Liu M., Liu Z., Zhou T., Liu Y., Cheng D. (2023). Network interpenetrating slow-release nitrogen fertilizer based on carrageenan and urea: A new low-cost water and fertilizer regulation carrier. Int. J. Biol. Macromol..

[167] Narayanaswamy R., Torchilin V.P. (2019). Hydrogels and their applications in targeted drug delivery. Molecules.

[168] Vashist A., Vashist A., Gupta Y.K., Ahmad S. (2014). Recent advances in hydrogel based drug delivery systems for the human body. J. Mater. Chem. B.

[169] Wang Y., Liu L., Zhu Y., Wang L., Yu D.-G., Liu L.-Y. (2023). Tri-Layer Core–Shell Fibers from Coaxial Electrospinning for a Modified Release of Metronidazole. Pharmaceutics.

[170] Hosseinzadeh S., Hosseinzadeh H., Pashaei S. (2019). Fabrication of nanocellulose loaded poly(AA-*co*-HEMA) hydrogels for ceftriaxone controlled delivery and crystal violet adsorption. Polym. Compos..

[171] Shin Y., Kim D., Hu Y., Kim Y., Hong I.K., Kim M.S., Jung S. (2021). pH-responsive succinoglycan-carboxymethyl cellulose hydrogels with highly improved mechanical strength for controlled drug delivery systems. Polymers.

[172] Bakravi A., Ahamadian Y., Hashemi H., Namazi H. (2018). Synthesis of gelatin-based biodegradable hydrogel nanocomposite and their application as drug delivery agent. Adv. Polym. Technol..

[173] Kapahi H., Khan N.M., Bhardwaj A., Mishra N. (2015). Implication of nanofibers in oral drug delivery. Curr. Pharm. Des..

[174] Zhou J., Dai Y., Fu J., Yan C., Yu D.-G., Yi T. (2023). Dual-Step Controlled Release of Berberine Hydrochloride from the Trans-Scale Hybrids of Nanofibers and Microparticles. Biomolecules.

[175] Zhou J., Wang L., Gong W., Wang B., Yu D.-G., Zhu Y. (2023). Integrating Chinese Herbs and Western Medicine for New Wound Dressings through Handheld Electrospinning. Biomedicines.

[176] Schneider A., Wang X., Kaplan D., Garlick J., Egles C. (2009). Biofunctionalized electrospun silk mats as a topical bioactive dressing for accelerated wound healing. Acta Biomater..

[177] Wu S., Liu J., Cai J., Zhao J., Duan B., Chen S. (2021). Combining electrospinning with hot drawing process to fabricate high performance poly (L-lactic acid) nanofiber yarns for advanced nanostructured bio-textiles. Biofabrication.

[178] Gao C., Zhang L., Wang J., Jin M., Tang Q., Chen Z., Cheng Y., Yang R., Zhao G. (2021). Electrospun nanofibers promote wound healing: Theories, techniques, and perspectives. J. Mater. Chem. B.

[179] Memic A., Abudula T., Mohammed H.S., Joshi Navare K., Colombani T., Bencherif S.A. (2019). Latest progress in electrospun nanofibers for wound healing applications. ACS Appl. Bio Mater..

[180] Liu Y., Li T., Han Y., Li F., Liu Y. (2021). Recent development of electrospun wound dressing. Curr. Opin. Biomed. Eng..

[181] Abrigo M., McArthur S.L., Kingshott P. (2014). Electrospun nanofibers as dressings for chronic wound care: Advances, challenges, and future prospects. Macromol. Biosci..

[182] Gao W., Sun L., Fu X., Lin Z., Xie W., Zhang W., Zhao F., Chen X. (2018). Enhanced diabetic wound healing by electrospun core–sheath fibers loaded with dimethyloxalylglycine. J. Mater. Chem. B.

[183] Choi J.S., Leong K.W., Yoo H.S. (2008). In vivo wound healing of diabetic ulcers using electrospun nanofibers immobilized with human epidermal growth factor (EGF). Biomaterials.

[184] Lalani R., Liu L. (2012). Electrospun Zwitterionic poly(sulfobetaine methacrylate) for nonadherent, superabsorbent, and antimicrobial wound dressing applications. Biomacromolecules.

[185] Gaydhane M.K., Kanuganti J.S., Sharma C.S. (2020). Honey and curcumin loaded multilayered polyvinylalcohol/cellulose acetate electrospun nanofibrous mat for wound healing. J. Mater. Res..

[186] Varshney N., Sahi A.K., Poddar S., Vishwakarma N.K., Kavimandan G., Prakash A., Mahto S.K. (2022). Freeze–thaw-induced physically cross-linked superabsorbent polyvinyl alcohol/soy protein isolate hydrogels for skin wound dressing: In Vitro and in vivo characterization. ACS Appl. Mater. Interfaces.

[187] Yu D.-G., Huang C. (2023). Electrospun Biomolecule-Based Drug Delivery Systems. Biomolecules.

[188] Wiegand C., Abel M., Ruth P., Hipler U.C. (2011). Superabsorbent polymer-containing wound dressings have a beneficial effect on wound healing by reducing PMN elastase concentration and inhibiting microbial growth. J. Mater. Sci. Mater. Med..

[189] Tarlton J.F., Munro H.S. (2013). Use of modified superabsorbent polymer dressings for protease modulation in improved chronic wound care. Wounds.

[190] Cipriano B.H., Banik S.J., Sharma R., Rumore D., Hwang W., Briber R.M., Raghavan S.R. (2014). Superabsorbent hydrogels that are robust and highly stretchable. Macromolecules.

[191] Zhu J., Marchant R.E. (2011). Design properties of hydrogel tissue-engineering scaffolds. Expert Rev. Med. Devices.

[192] Garg T., Singh O., Arora S., Murthy R.S.R. (2012). Scaffold: A novel carrier for cell and drug delivery. Crit. Rev. Ther. Drug Carr. Syst..

[193] Wojcik M., Kazimierczak P., Benko A., Palka K., Vivcharenko V., Przekora A. (2021). Superabsorbent curdlan-based foam dressings with typical hydrocolloids properties for highly exuding wound management. Mater. Sci. Eng. C Mater. Biol. Appl..

[194] Chen W., Ma J., Zhu L., Morsi Y., Ei-Hamshary H.A., Al-Deyab S.S., Mo X. (2016). Superelastic, superabsorbent and 3D nanofiber-assembled scaffold for tissue engineering. Colloids Surfaces B Biointerfaces.

[195] Yang X., Yang D., Zhu X., Nie J., Ma G. (2019). Electrospun and photocrosslinked gelatin/dextran–maleic anhydride composite fibers for tissue engineering. Eur. Polym. J..

[196] Sartore L., Pandini S., Baldi F., Bignotti F., Di Landro L. (2017). Biocomposites based on poly(lactic acid) and superabsorbent sodium polyacrylate. J. Appl. Polym. Sci..

[197] Mahmoodzadeh A., Moghaddas J., Jarolmasjed S., Kalan A.E., Edalati M., Salehi R. (2021). Biodegradable cellulose-based superabsorbent as potent hemostatic agent. Chem. Eng. J..

[198] Chen W., Chen S., Morsi Y., El-Hamshary H., El-Newhy M., Fan C., Mo X. (2016). Superabsorbent 3D scaffold based on electrospun nanofibers for cartilage tissue engineering. ACS Appl. Mater. Interfaces.

[199] Rnjak-Kovacina J., Weiss A.S. (2011). Increasing the pore size of electrospun scaffolds. Tissue Eng. Part B Rev..

[200] Chen S., Wang H., McCarthy A., Yan Z., Kim H.J., Carlson M.A., Xia Y., Xie J. (2019). Three-dimensional objects consisting of hierarchically assembled nanofibers with controlled alignments for regenerative medicine. Nano Lett..

[201] Fei J., Tang T., Zhou L., He H., Ma M., Shi Y., Chen S., Wang X. (2023). High-Toughness and Biodegradable Superabsorbent Hydrogels Based on Dual Functional Crosslinkers. ACS Appl. Polym. Mater..

[202] Duan H., Chen H., Qi C., Lv F., Wang J., Liu Y., Liu Z., Liu Y. (2024). A novel electrospun nanofiber system with PEGylated paclitaxel nanocrystals enhancing the transmucus permeability and in situ retention for an efficient cervicovaginal cancer therapy. Int. J. Pharm..

[203] Kang S., Hou S., Chen X., Yu D.-G., Wang L., Li X., Williams G.R. (2020). Energy-Saving Electrospinning with a Concentric Teflon-Core Rod Spinneret to Create Medicated Nanofibers. Polymers.

[204] Chen S., Zhou J., Fang B., Ying Y., Yu D., He H. (2023). Three EHDA Processes from a Detachable Spinneret for Fabricating Drug Fast Dissolution Composites. Macromol. Mater. Eng..

[205] Chen X., Liu Y., Liu P. (2023). Electrospun Core–Sheath Nanofibers with a Cellulose Acetate Coating for the Synergistic Release of Zinc Ion and Drugs. Mol. Pharm..

[206] Qian C., Liu Y., Chen S., Zhang C., Chen X., Liu Y., Liu P. (2023). Electrospun core–sheath PCL nanofibers loaded with nHA and simvastatin and their potential bone regeneration applications. Front. Bioeng. Biotechnol..

[207] Cao X., Chen W., Zhao P., Yang Y., Yu D.-G. (2022). Electrospun Porous Nanofibers: Pore−Forming Mechanisms and Applications for Photocatalytic Degradation of Organic Pollutants in Wastewater. Polymers.

[208] Bai Y., Liu Y., Lv H., Shi H., Zhou W., Liu Y., Yu D.-G. (2022). Processes of Electrospun Polyvinylidene Fluoride-Based Nanofibers, Their Piezoelectric Properties, and Several Fantastic Applications. Polymers.

[209] Lv Q., Ma X., Zhang C., Han J., He S., Liu K., Jiang S. (2024). Nanocellulose-based nanogenerators for sensor applications: A review. Int. J. Biol. Macromol..

[210] Yu D.G., Zhou J. (2024). Electrospun Multi-Chamber Nanostructures for Sustainable Biobased Chemical Nanofibers. Next Mater..

[211] Li J., Du Q., Wan J., Yu D.-G., Tan F., Yang X. (2024). Improved synergistic anticancer action of quercetin and tamoxifen citrate supported by an electrospun complex nanostructure. Mater. Des..

